# Profiling of somatic mutations and fusion genes in acute myeloid leukemia patients with *FLT3*-ITD or *FLT3*-TKD mutation at diagnosis reveals distinct evolutionary patterns

**DOI:** 10.1186/s40164-021-00207-4

**Published:** 2021-04-09

**Authors:** Wei Guan, Lei Zhou, Yan Li, Erna Yang, Yangyang Liu, Na Lv, Lin Fu, Yi Ding, Nan Wang, Nan Fang, Qian Liu, Binan Wang, Fuwei Li, Juan Zhang, Maoquan Wang, Lili Wang, Yu Jing, Yonghui Li, Li Yu

**Affiliations:** 1 Department of Hematology, Chinese PLA General Hospital, 28 Fuxing Road, Beijing, 100853 China; 2 Department of Hematology, Peking University Third Hospital, 49 North Garden Road, Beijing, 100191 China; 3 Department of Hematology and Oncology, Shenzhen Key Laboratory of Precision Medicine for Hematological Malignancies, Carlson International Cancer Center, Shenzhen University General Hospital, Shenzhen University Health Science Center, 1098 Xueyuan AVE, Shenzhen, 518060 China; 4 Beijing USCI Medical Laboratory Co., Ltd, Beijing, China; 5 Department of Hematology, The Second Affiliated Hospital of Guangzhou Medical University, Guangzhou, 510260 China; 6 School of Medicine, Nankai University, 94 Weijin Road, Tianjin, 300071 China

**Keywords:** Acute myeloid leukemia, *FLT3*-ITD, *FLT3*-TKD, *TET2*, Next-generation sequencing

## Abstract

**Background:**

The receptor tyrosine kinase *FLT3* with internal tandem duplications within the juxtamembrane domain (*FLT3*-ITD) is a poor prognostic factor; however, the prognostic significance of missense mutation in the tyrosine kinase domain (*FLT3*-TKD) is controversial. Furthermore, the accompanying mutations and fusion genes with *FLT3* mutations are unclear in acute myeloid leukemia (AML).

**Methods:**

We investigated *FLT3* mutations and their correlation with other gene mutations and gene fusions through two RNA-seq based next-generation sequencing (NGS) method and prognostic impact in 207 de novo AML patients.

**Results:**

*FLT3*-ITD mutations were positive in 58 patients (28%), and *FLT3*-TKD mutations were positive in 20 patients (9.7%). *FLT3-ITD* was associated with a higher white blood cell count (WBC, mean 72.9 × 10^9^/L vs. 24.2 × 10^9^/L, *P* = 0.000), higher bone marrow blasts (mean 65.9% vs. 56.0%, *P* = 0.024), and NK-AML (normal karyotype) (64.8% vs. 48.4%, *P* = 0.043). *NPM1* and *DNMT3A* mutations were enriched in *FLT3*-ITD (53.5% vs. 15.3%, *P* = 0.000; 34.6% vs. 13%, *P* = 0.003). However, the mutations of *CEBPA* were excluded in *FLT3*-AML (3.8% vs. 0% vs. 19.8%, *P* = 0.005). Mutations of *Ras* and *TP53* were unlikely associated with *FLT3*-ITD (1.9% vs. 20.6%, *P* = 0.006; 0% vs. 6.1%, *P* = 0.04). The common fusion genes (> 10%) in *FLT3*-ITD had *MLL-*rearrangement and *NUP98*-rearrangement, while the common fusion genes in *FLT3*-TKD had *AML1-ETO* and *MLL*-rearrangement. Two novel fusion genes *PRDM16-SKI* and *EFAN2-ZNF238* were identified in FLT3-ITD patients. Gene fusions and *NPM1* mutation were mutually excluded in *FLT3*-ITD and *FLT3*-TKD patients. Their patterns of mutual exclusivity and cooperation among mutated genes suggest that additional driver genetic alterations are required and reveal two evolutionary patterns of *FLT3* pathogenesis. Patients with *FLT3*-ITD had a lower CR (complete remission) rate, lower 3-year OS (overall survival), DFS (disease-free survival), and EFS (event-free survival) compared to *FLT3*_wt_AML. NK-AML with *FLT3*-ITD had a lower 3-year OS, DFS, and EFS than those without, while *FLT3*-TKD did not influence the survival in whole cohort and NK-AML. Besides, we found that *FLT3*-ITD/*TET2* bimutation defined a poor prognostic subgroup.

**Conclusions:**

Our study offers deep insights into the molecular pathogenesis and biology of AML with *FLT3*-ITD and *FLT3*-TKD by providing the profiles of concurrent molecular alterations and the clinical impact of *FLT3*-ITD and *FLT3*-TKD on AML patients.

## Introduction

Acute myeloid leukemia (AML) is a heterogeneous hematological malignancy accompanied by complex molecular genetic abnormalities with an increasing incidence in the globe [1–3]. *FLT3* mutation is one of the most common mutations in AML. *FLT3* with internal tandem duplications within the juxtamembrane domain (*FLT3*-ITD) is present in 20–30% of AML patients, and a missense mutation in the tyrosine kinase domain (*FLT3*-TKD) accounts for about 10% of AML [4, 5]. *FLT3*-ITD alone does not trigger leukemia, indicating that other drivers are needed for pathogenies [6, 7]. *FLT3*-ITD with additional *NPM1* mutation [8], *AML1-ETO* fusion gene [9], *NUP98* fusion [10, 11], *CBFβ-SMMHC* fusion gene [12], and *TET2* deletion [13, 14] can cause leukemia. The complex pathogenic mechanism and heterogeneous clinical features of *FLT3*-ITD necessitate comprehensive molecular profiling. Owing to the low incidence of *FLT3*-TKD mutation, neither the prognostic significance is clear nor the accompanying molecular alterations.

Gene fusion is a very important pathogenic mechanism, and each fusion gene has its unique clinical manifestations. *BCR-ABL* resulting from t(9;22) in chronic myelogenous leukemia (CML) is a classic example [15]. Since the discovery of *BCR/ABL*, CML entered the era of targeted treatment, significantly improving the survival [16, 17]. Fusion genes are effective targets for diagnosis, prognosis, therapy, and minimal residual disease (MRD) monitoring in hematological cancers [18]. The detection of common fusion genes with clinical significance has become a routine practice today. The next-generation sequencing (NGS) technique has much more advantages in detecting fusion genes. RNA-seq based NGS can provide information about the structure and transcript level of fusion genes. Its technical advance makes the global identification of fusion transcripts possible [19]. Targeted NGS sequencing for fusion genes in *FLT3* mutant AML has not been reported before. In this study, coexisting gene mutations and fusion genes of *FLT3*-ITD and *FLT3*-TKD mutation in AML patients by NGS were analyzed to better understand this disease.

## Patients and methods

### Patients and study design

A total of 207 patients (older than 14 years) with newly diagnosed AML (non-M3) admitted to the hospital from August 2009 to October 2017 were analyzed. According to the cytogenetically defined MRC criteria, 23 patients of this cohort were assigned to the favorable-prognostic-risk group, 151 to the intermediate-prognostic-risk group, and 23 to the poor-prognostic-risk group. In detail, 103 patients had a normal karyotype; six patients had a complex aberrate karyotype; 23 patients had a t(8;21); eight patients had a 11q23 rearrangement; 57 patients had other aberrant karyotypes. Cytogenetics of 10 patients were not available because of analysis failure or missing information. The cohort included 115 male and 94 female patients. The median age was 45.4 years (ranging from 14 to 76 years). Incidence of *FLT3* mutations and correlation with other recurrent mutations and fusions in AML were evaluated in this cohort.

Fifty-eight cases were *FLT3*-ITD positive (28%), and 20 cases were *FLT3*-TKD mutation-positive (9.7%), four of which carried both mutations. *FLT3*-ITD analysis was based on DNA capture sequencing. The filtered reads were compared to the reference genome sequence (HG19, NCBI Built 37) using Burrows–Wheeler alignment (BWA), and the insertion and deletion of *FLT3* region were detected using Pindel (0.2.4) software to detect *FLT3*-ITD mutation. The variation was annotated using ANNOVAR. The reads were aligned using BWA tool to human genomic reference sequences (HG19, NCBI built 37). To identify SNPs and INDELs, GATK was performed with recommended parameters; Pindel (0.2.4) was performed to identify the *FLT3*-ITD. *FLT3*-ITD was simultaneously verified by Sanger sequencing. 52/58 *FLT3*-ITD patients were detected by two targeted NGS for mutations and fusions. Four *FLT3*-TKD patients co-occurring with *FLT3*-TKD were assigned to the *FLT3*-ITD group; the other 16 *FLT3*-TKD patients were also detected by NGS for mutations and fusion genes assigned as *FLT3*-TKD group. The other 133 *FLT3* wild-type AML (*FLT3*_wt_AML) patients were detected by Sanger sequencing for molecular mutation analyses only. The study was designed following the Declaration of Helsinki and approved by the institutional review board of PLA general hospital.

### Therapy

Forty-six *FLT3*-ITD patients, 14 *FLT3*-TKD patients, and 113 *FLT3*_wt_ patients completed two cycles of induction, and they were evaluated for treatment response. TKI inhibitor was applied in the induction regimen for three patients with *FLT3*-ITD [Sunitinib + AA (n = 1) and Sorafenib + FLAG (n = 2)]. Consolidation therapy after complete remission (CR) was administered to 29 patients in the *FLT3*-ITD group, 10 patients in the *FLT3*-TKD group, and 93 *FLT3*_wt_ patients. Sorafenib was administrated in consolidation chemotherapy for one patient with *FLT3*-ITD and after HSCT for one patient with *FLT3*-ITD to prevent relapse. In total, 13 patients with *FLT3*-ITD, 5 with *FLT3*-TKD, and 47 *FLT3* wild type received SCT in CR1. Treatment options for chemotherapy and stem cell transplantation were not significantly different among the three groups (*P* = 0.865). The treatment flow diagram is shown in Additional file 1: Fig. S1. The data of two *FLT3*-ITD patients and one *FLT3*_wt_ patient were cancelled for survival analysis due to the loss of follow-up.

### Library preparation and NGS

The method and gene panel of NGS for mutation detection in AML are previously reported [20]. The NGS for fusion gene detection is based on targeted RNA-seq. In brief, RNA was extracted from patient samples using the Tempus Spin RNA Isolation Kit (Life) following the manufacturer’s instructions. The RNA quality [RNA integrity number (RIN)] was assessed using an Agilent 2100 Bioanalyzer and RNA 6000 Nano Kit and quantified using a Qubit^®^ 3.0 fluorometer and Qubit RNA HS Assay Kit. Samples with a total of 1500 ng RNA and RIN ≥ 4.1 were used as the input for the next library preparation. Briefly, the first- and second-strand complementary DNA (cDNA) was synthesized using a PrimeScript Double Strand cDNA Synthesis Kit (Takara). Double-stranded cDNA was then cleaned with Agencourt AMPure XP beads (Beckman Coulter) and subjected to end-repair, adenylation, and ligation using a universal barcode adapter, subjected, and amplified by seven cycles to generate the mid-libraries. The target genes were captured with a specific panel from the mid-libraries, amplified, and then sequenced. Paired-end, 101 bp sequencing was performed using a HiSeq 2500 (Illumina) instrument in the Rapid Run mode. The sequence was aligned to the reference sequence using Hisat2 (2.0.3). FusionMap software was used to detect the fusion genes, and Blacklist filtering was used to remove the ribosomal genes, mitochondrial genes, and fusions of pseudogenes, as well as the fusions between gene families and homologous genes. The targeted fusion genes are shown in Additional file 4: Table S1.

### Statistics method

The data were analyzed and processed using GraphPad 7.0 software. The measurement data conforming to normal distribution were compared using a Student’s t-test and variance analysis. The mean value of measurement data that did not conform to normal distribution was compared using a rank-sum test. The frequency of counting data was expressed in %, and the rates were compared by conducting a χ^2^ test. The survival curve was tested using the log-rank method. Overall survival (OS) was calculated from diagnosis to death. Disease-free survival (DFS) was calculated from the first CR to relapse or death, and patients who did not achieve CR were excluded. Event-free survival (EFS) was calculated from diagnosis to relapse or death of any cause. A statistical difference was considered at *P* < 0.05.

## Results

### Clinical associations

The frequency of *FLT3*-ITD and *FLT3*-TKD mutation was 28%, and 9.7%, respectively. The general characteristics of *FLT3*-ITD AML, *FLT3*-TKD AML, and *FLT3*_wt_ AML are shown in Table 1. The count of white blood cell (WBC) and the proportion of blasts in the bone marrow of *FLT3*-ITD group was higher than that of *FLT3*_wt_AML group (*P* = 0.000 and *P* = 0.024, respectively). The count of WBC of *FLT3*-TKD group was also higher than that of *FLT3*_wt_AML group, *P* = 0.008. There was no significant difference in the proportion of bone marrow blasts between the *FLT3*-TKD group and *FLT3*_wt_AML group, *P* > 0.05. *FLT3*-ITD was associated with normal karyotype (64.8% vs. 48.4%, *P* = 0.043); in contrast, *FLT3*-TKD showed no difference in karyotype distribution compared to *FLT3*_wt_ AML, (40% vs. 48.4%, *P* > 0.05).

**Table 1:** Clinical, cytogenetics and molecular genetics characteristic of 207 analyzed AML patients

Parameter	*FLT3*-ITD (n = 58)	*FLT3*-TKD (n = 16)	*FLT3*_wt_AML (n = 133)	*P* value^a^
Male	23 (39.7)	12 (75)	80 (60.2)	*0.009*
Age	48 (14–73)	41 (14–76)	45 (15–76)	0.367
WBC at diagnosis, × 10^9^/L	72.9 (2.3–405.1)	68.2 (1.8–251.1)	24.2 (0.57–311.0)	*0.000*
Blasts in BM, %	65.9 (22.0–95.6)	55.5 (30.8–94.0)	56.0 (14.4–94.5)	*0.040*
FAB subtype, n (%)				0.983
M0	0	0	0	
M1	3 (5.2)	1 (6.3)	4 (3.0)	
M2	16 (27.6)	5 (31.3)	38 (28.6)	
M4	20 (34.5)	6 (37.5)	41 (30.8)	
M5	14 (24.1)	4 (25.0)	36 (27.1)	
M6	2 (34)	0	5 (3.8)	
Unclassified	1 (1.7)	0	6 (4.5)	
Secondary-AML	2 (3.4)	0	3 (2.3)	
Cytogenetics, n (%) (n = 197)				
Normal karyotypes	35 (64.8)	6 (40.0)	62 (48.4)	*0.079*
Aberrant karyotypes	19 (35.2)	9 (60.9)	66 (51.6)	
Gene Mutation^c^, n (%)				
* NPM1*	28 (53.8)^&^	4 (25)	20 (15.3)	*0.000*
* DNMT3A*	18 (34.6)^&^	4 (25)	17(13.0)	*0.003*
* RUNX1*	1 (1.7)	1 (6.3)	8 (6.1)	0.492
* KIT*	3 (5.8)	0 (0)	6 (4.6)	0.623
* RAS*	1 (1.9)^&^	1 (6.3)	27 (20.6)	*0.003*
* PTPN11*	5 (9.6)	1 (6.3)	6 (6.3)	0.435
* TET2*	6 (11.5)	1 (6.3)	10 (7.6)	0.656
* IDH1/2*	5 (19.6)	3 (18.8)	20 (15.3)	0.522
* CEBPA*	2 (3.8)^&^	0 (0)	26 (19.8)	*0.005*
* ASXL1*	2 (3.8)	2 (12.5)	13 (9.9)	0.348
* TP53*	0 (0)^&^	1/16 (6.3)	8 (6.1)	*0.189*
Methylation-related genes^b^	23 (44.2)	6 (37.5)	42 (32.1)	0.297
Number of mutations	3.2 (1–7)^&^	3.6 (1–6)	2.7 (0–8)	*0.022*
CR after two cycles of induction	29/46 (63)	10/14 (71.4)	100/113 (88.5)	*0.001*
Consolidation in CR1				
CT SCT	16 (55.2) 13 (44.8)	5 (50) 5 (50)	6 (49.5) 47 (50.5)	0.865
Three-year OS (%)	36 ± 9.1	65.6 ± 15.1	50.6 ± 7	*0.020*
Three-year EFS (%)	27.2 ± 8.1	55.9 ± 16.2	40.5 ± 6.5	*0.005*

Italic values indicate significance of *P* value (*P* < 0.05)

*WBC* white blood count, *BM* bone marrow, *FAB* French–America–British, *CR* complete remission, *CT* chemotherapy, *SCT* stem cell transplantation

^a^*P*-values for categorical variables are from chi-square test, P-values for continuous variables are from the ANOVA test

^b^Methylation related gene included *DNMT3A*, *IDH1/2*, and *TET2*

^c^52 *FLT3*-ITD, 16 *FLT3*-TKD and 131 *FLT3* wildtype patients were analyzed for gene mutations

^#^p value for frequency of favorable, intermediate and unfavorable karyotype in three groups

^&^*P* value < 0.05 between the *FLT3*-ITD group and *FLT3*_wt_ group

**Fig. 1:**
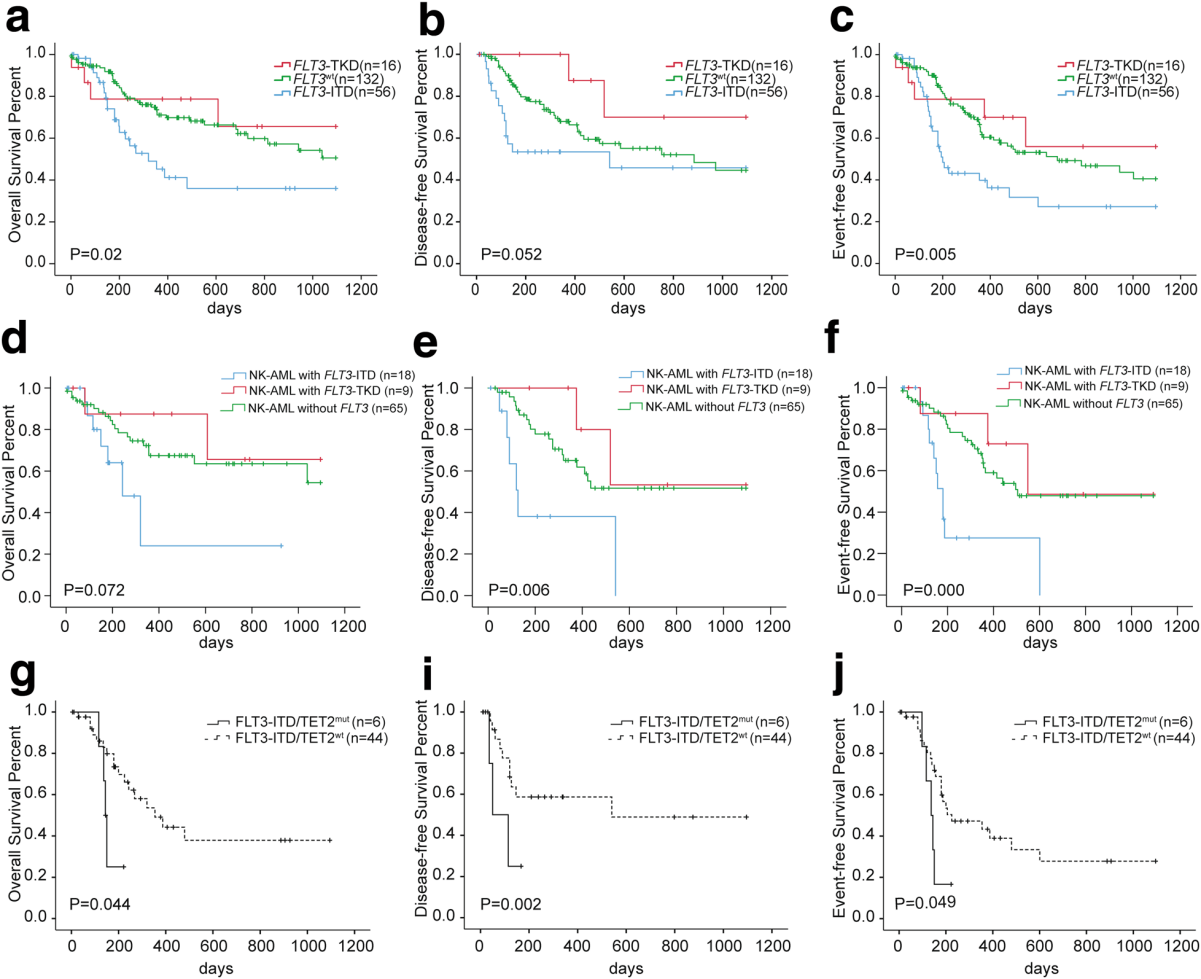
OS (**a**), DFS (**b**), and EFS (**c**) curve of *FLT3*-TKD (n = 16), *FLT3*-ITD (n = 56), and *FLT3* wild type (n = 132) AML patients; OS (**d**), DFS (**e**), and EFS (**f**) curve of normal karyotype AML patients with (n = 18) or without (n = 74) *FLT3*-ITD mutation; OS (**h**), DFS (**i**), and EFS (**g**) curve of *FLT3*-ITD AML patients with (n = 6) or without (n = 44) *TET2* mutation

The CR rate after two cycles of induction of *FLT3*-ITD group was lower than that of *FLT3*_wt_AML group (63% and 88.5%, respectively, *P* = 0.000). The CR rate of *FLT3*-TKD patients was is 71.4%, not significantly different from that of the *FLT3*_wt_AML group, *P* = 0.077. The *FLT3*-ITD group had a lower three-year OS, DFS, and EFS than those of *FLT3*-TKD group and *FLT3*_wt_AML group (36% ± 9.1% vs. 65.6% ± 15.1% vs. 50.6% ± 4.6%, respectively, *P* = 0.02; 45.8% ± 10.8% vs. 70% ± 18.2% vs. 44.6% ± 7.4%, respectively, *P* = 0.052; 27.2% ± 8.1% vs. 55.9% ± 16.2% vs. 40.5% ± 6.5%, respectively, *P* = 0.005) (Fig. 1a–c). The three-year OS, DFS, and EFS of *FLT3*-ITD group, *FLT3*-TKD group, and *FLT3*-ITD_wt_ group in normal karyotype (NK)-AML are shown in Fig. 1d–f. No difference was observed between *FLT3*-TKD group and *FLT3*_wt_ group in three-year OS, DFS, and EFS in NK-AML (65.5% ± 20.9% vs. 54.4% ± 10.5%, *P* = 0.538; 53.3% ± 24.8% vs. 51.7% ± 8.4%, *P* = 0.43; 48.6% ± 22.7% vs. 47.9% ± 7.6%, *P* = 0.557). *FLT3*-ITD could stratify the outcomes of NK-AML patients (24% ± 19% vs. 54.4% ± 10.5%, *P* = 0.035; 0% vs. 51.7% ± 8.4%, *P* = 0.004; 0% vs. 47.9% ± 7.6%, *P* = 0.000). Furthermore, co-occurring *TET2* mutation impaired the 3-year OS, DFS, and EFS of patients with *FLT3*-ITD (37.9% ± 10.3% vs. 25% ± 20.4%, *P* = 0.044; 48.9% ± 12.6% vs. 16.7% ± 15.2%, *P* = 0.002; 27.8% ± 9.2% vs. 16.7% ± 15.2%, *P* = 0.049) (Fig. 1g–i).

### Associations with fusion genes

**Fig. 2:**
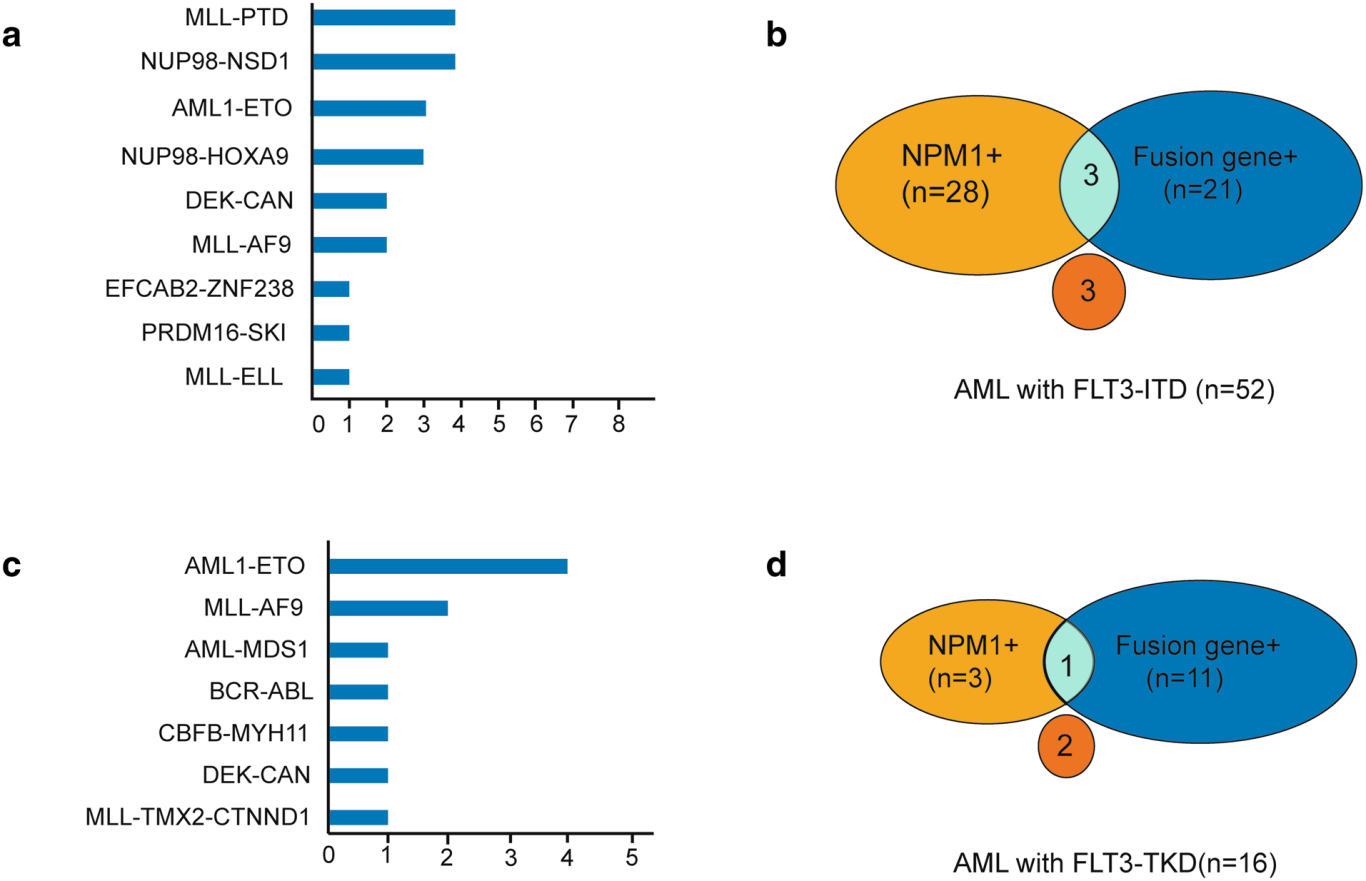
Relationship between gene mutations and fusion genes of *FLT3*-ITD and *FLT3*-TKD AML. **a**, **b** Represent fusion genes by targeted NGS and its exclusive relationship with *NPM1* mutation in *FLT3*-ITD positive AML (n = 60). **c**, **d** Represent fusion genes by targeted NGS and its exclusive relationship with *NPM1* mutation in *FLT3*-TKD positive AML (n = 16)

Among the 52 patients with *FLT3*-ITD, 21 had fusion genes, and the incidence of fusion genes was 40.4% (Fig. 2a, b). The most common fusion genes of *FLT3*-ITD AML included seven *MLL*-rearranged (13.5%) (four *MLL*-PTD, two *MLL-AF9*, and one *MLL-ELL)* and seven *NUP98*-rearranged (13.5%) (four *NUP98-NSD1* and three *NUP98-HOX9A*). Other recurrent fusions included three with *AML1-ETO* and two with *DEK/CAN*. One case with *PRDM16-SKI* and one case with *EFAN2-ZNF238* fusion gene are reported for the first time.

Among the 16 *FLT3*-TKD mutant AML patients, 11 cases had fusion genes (four cases with *AML1-ETO*, two cases with *MLL-AF9*, one case with *AML1-MDS1*, one case with *DEC-CAN*, one case with *BCR-ABL*, one case with *CBFB-MYH11*, and one case with *MLL-TMX2 -CTNND1*) (Fig. 2c, d). The frequency of fusion genes of *FLT3*-TKD group was higher than that of *FLT3*-ITD group (68.75% vs. 40.4%, *P* = 0.014). Among patients with *FLT3*-TKD, 11 patients were associated with fusion genes, as described in the manuscript, four cases with *AML1-ETO*, two cases with *MLL-AF9*, one case with *AML1-MDS1*, one case with *DEC-CAN*, one case with *BCR-ABL*, one case with *CBFB-MYH11*, and one case with *MLL-TMX2-CTNND1*. Both patients with *MLL* rearrangement were refractory to induction therapy and died of disease progression. The prognosis of four patients with *AML1-ETO* was relatively good, among which two patients achieved long-term survival through chemotherapy and transplantation. Two patients with *AML1-ETO* relapsed and were salvaged by allo-HSCT, one of who relapsed and died after salvaged transplantation, and the other patient achieved long-term survival after salvaged transplantation. The patient with *BCR/ABL1* fusion died during induction. The remaining patients with *DEC-CAN*, *AML-MDS1*, and *CBFC-MYH11* achieved long-term survival. Among them, the patient with *DEC-CAN* received allogeneic transplantation, the patient with *AML-MDS1* received autograft, and the patient with *CBFB-MYH11* received chemotherapy as consolidation after remission.

Fusion genes and chromosome karyotype characteristics are shown in Table 2. NGS was efficient in the detection of gene fusions, especially in the rare fusions or *MLL* translocation partner genes. Cytogenetics analysis failed to detect all *NUP98-NSD1* and four of five *MLL* fusions. Furthermore, one *MLL/MLLT3* and one *MLL/TMX2-CTNND1* were detected by only NGS. In normal karyotypes cases and negative cases by routine PCR, NGS identified seven fusion genes including four with *NUP98-NSD1*, four with *AML/MDS1*, one with *PRDM16/SKI*, one with *EFCAB2/ZNF238*, and one with *MLL/MLLT3*. One *MLL* fusion with rare translocation partner genes *TMX2/CTNND1* was detected by NGS, while without providing information by karyotype analysis (Table 2).

**Table 2:** Fusion genes by NGS and PCR and Chromosome karyotype analysis in *FLT3* mutant AML

Fusion gene by NGS	Fusion gene by PCR	Chromosome karyotype	*FLT3* mutation
*AML1/ETO*	*AML1/ETO*	46, XY, t(8;21)(q22;q22)[20]	*FLT3*-ITD, *FLT3*-TKD
*AML1/ETO*	*AML1/ETO*	46, X, -X,t(8;21)(q22;q22), del(9)(q22)[9]/46,XX,t(8;21)(q22;q22)[11]	*FLT3*-ITD
*AML1/ETO*	*AML1/ETO*	45, X, -X,t(8;21)[20]	*FLT3*-ITD
*MLL-PTD*	*MLL-PTD*	46, XY[20]	*FLT3*-ITD
*MLL-PTD*	*MLL-PTD*	46, XY[20]	*FLT3*-ITD
*MLL-PTD*	*MLL-PTD*	46, XX[20]	*FLT3*-ITD
*MLL-PTD*	*MLL-PTD*	47, XY, + 8?[10]/46, XY[10]	*FLT3*-ITD
*MLL/AF9*	*MLL/AF9*	46, XX,?der(2)(q11),inc[1] /46,XX[28]/hypodiploid [4] (44–45)	*FLT3*-ITD
*MLL/AF9*	*MLL/AF9*	47, XY, + 8[7]	*FLT3*-ITD
*MLL/ELL*	*MLL/ELL*	46, XX, t(11;19)(q23; q13)[10]	*FLT3*-ITD
*NUP98/HOXA9*	*NUP98/HOXA9*	NA	*FLT3*-ITD
*NUP98/HOXA9*	*NUP98/HOXA9*	46, XX[20]	*FLT3*-ITD
*NUP98/HOXA9*	*NUP98/HOXA9*	NA	*FLT3*-ITD
*NUP98-NSD1*	*–*	46, XX[20]	*FLT3*-ITD
*NUP98-NSD1*	*–*	47, XX, + 6[14]/46, XX[6]	*FLT3*-ITD
*NUP98-NSD1*	*–*	46, XY[20]	*FLT3*-ITD
*NUP98-NSD1*	*–*	46, XY[25]	*FLT3*-ITD
*DEK/CAN*	*DEK/CAN*	46, XX[20]	*FLT3*-ITD
*DEK/CAN*	*DEK/CAN*	46, XY, ?t(6;9)(p23;34)[10]/46, XY,?t(6;9)(p23;q34),?del(8)(q21)[11]/46,XY[1]	*FLT3*-ITD
*PRDM16-SKI*	*–*	46, XX[20]	*FLT3*-ITD
*EFCAB2-ZNF238*	*–*	46, XX [20]	*FLT3*-ITD
*BCR/ABL*	*BCR/ABL*	NA	*FLT3*-TKD
*AML1/ETO*	*AML1/ETO*	45, X, -Y, t(8;21)(q22;q22)[22]	*FLT3*-TKD
*AML1/ETO*	*AML1/ETO*	46, XY,t(8;21)(q22;q22)[26]/46,XY[1]	*FLT3*-TKD
*AML1/ETO*	*AML1/ETO*	46, XX, t(8;21)(q22;q22)[20]	*FLT3*-TKD
*AML1/ETO*	*AML1/ETO*	45,X,?Xq-,?8q-,-22[1]/43,X,?Xq-,-8,-10,-22[1]/45,X,-X[1]/47,XX, + mar[1]/40,-X,-X,-11,-21,-22, + mar[1]/46,XX[4]	*FLT3*-TKD
*MLL/MLLT3*	*–*	46, XX[20]	*FLT3*-TKD
*DEK/CAN*	*DEK/CAN*	47, XY, chtb(4)(?q31),? + 9,-15,inc[1]/46, XY[27]/hypodiploid [2] (44–45)	*FLT3*-TKD
*AML/MDS1*	*–*	46, XY [20]	*FLT3*-TKD
*SLC45A3/ELK4*	*–*	47, XY, + 8[7]	*FLT3*-TKD
*MLL/TMX2-CTNND1*	*–*	42–47,XY, + 3,del(3)(p13),del(3)(q13),-4,?add(4)(q35),-8,-11,dic(11;?)(q25;?),-16,-17,-18,-19,-20, + r, + mar1, + mar2, + mar3,inc[cp22]/46,XY[1]	*FLT3*-TKD
*CBFB/MYH11*	*CBFB/MYH11*	47, XY, + 22[2]/46, XY[23]	*FLT3*-TKD

NGS, next generation sequencing; PCR, polymerase chain reaction

### Associations with other molecular mutations

**Fig. 3:**
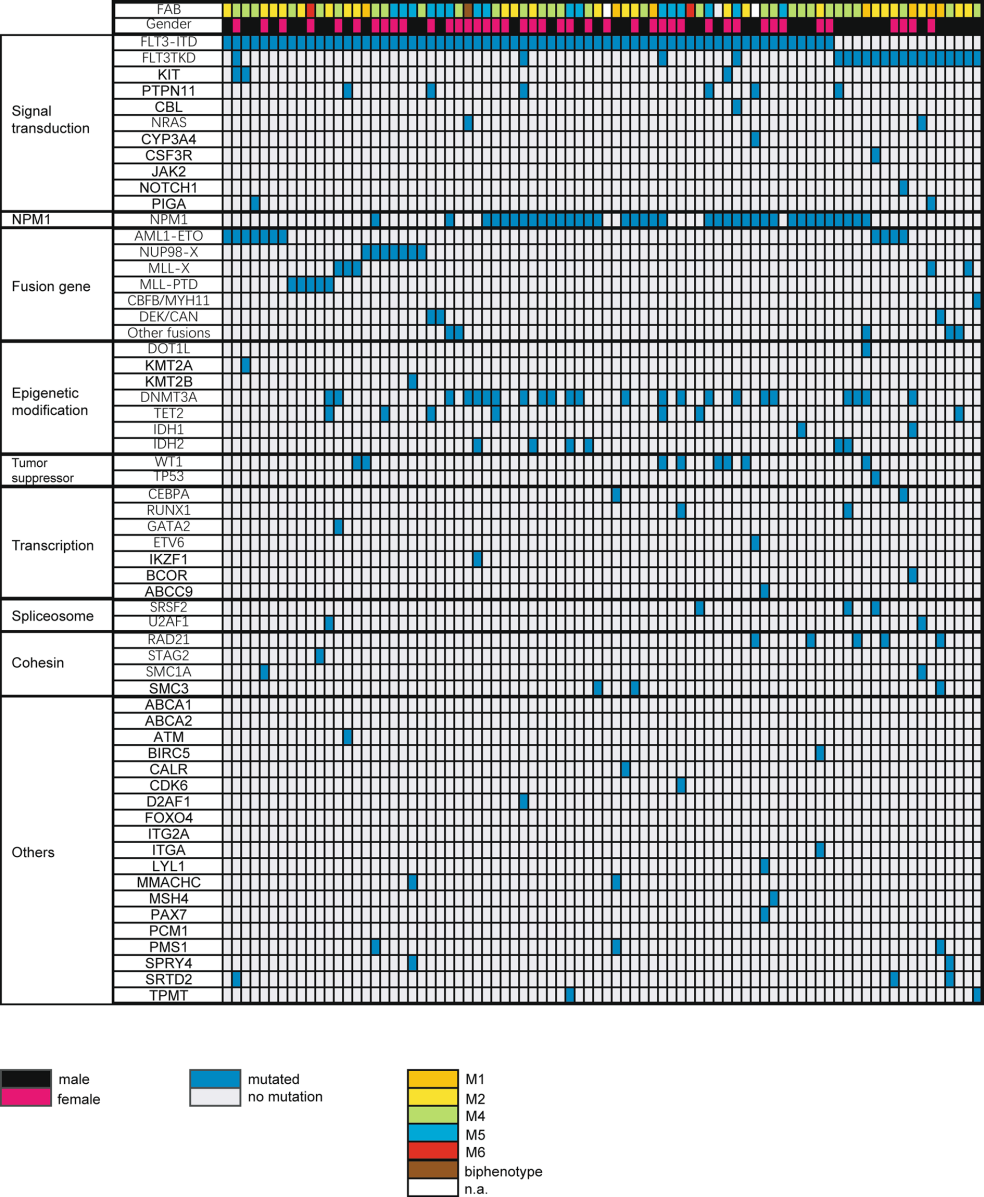
Distribution of somatic mutations and fusion genes in 82 AML patients with *FLT3*-ITD and *FLT3*-TKD. Each column displays an individual sample. White highlights in the top FAB subtype indicate that the information is not available (n.a.). Blue highlights indicate the presence of a gene mutation; grey highlights indicate wild-type status. *CEBPA* mutation is an allele double mutation in this panel. Mutated genes are clustered according to their pathways or family

**Fig. 4:**
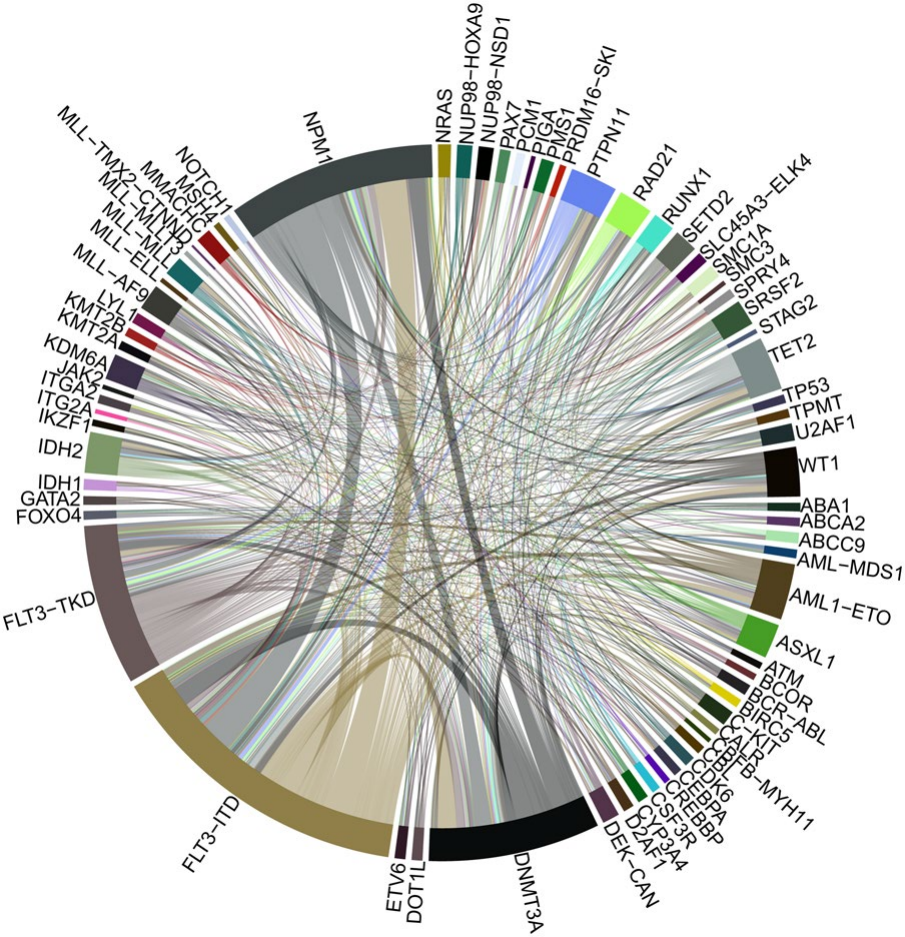
Circos of mutated genes and gene fusions in AML patients with *FLT3* mutation. Ribbon widths are proportional to the frequency of a molecular event

The mutation data were available in subcohorts as follows: 52 *FLT3*-ITD, 16 *FLT3*-TKD, and 131 *FLT3*_wt_ patients. *NPM1* and *DNMT3A* were concomitantly observed together with *FLT3*-ITD (Table 1; Figs. 3, 4). The frequency of *NPM1* mutation was 53.8% in *FLT3*-ITD AML, higher than that of *FLT3*_wt_AML group (15.3%), *P* = 0.000. The second frequent mutation was *DNMT3A*, with a frequency of 34.6%, significantly higher than that of *FLT3*_wt_AML group, *P* = 0.001. However, the mutation in *CEBPA* and *Ras* were highly infrequent in *FLT3*-ITD AML (2/52 (3.8%) vs. 26/131 (19.8%), *P* = 0.007; 1/52(1.9%) vs. 27/131(20.6%), *P* = 0.002). *RAS* mutations in *FLT3*-ITD (n = 1) and *FLT3*-TKD (n = 1) were both *NRAS* mutation. *RAS* isoforms (n = 27) in *FLT3*_wt_ patients were *NRAS* in 22 cases, *KRAS* in four cases, and both *NRAS* and *KRAS* in one case. Further, *TP53* mutations were mutually exclusive of *FLT3*-ITD (0/58 vs. 8/131, *P* = 0.004). The average number of mutations in *FLT3*-ITD AML was 3.7, higher than that in *FLT3*_wt_AML (average number = 2.7, *P* = 0.011).

In contrast to *FLT3*-ITD, no significant difference was observed in the incidence of *NPM1*, *DNMT3A*, and *RAS* mutations between *FLT3*-TKD group and *FLT3*_wt_AML group. Mutations of *CEBPA* were also excluded in *FLT3*-TKD mutant patients (0/16 vs. 26/131, *P* = 0.05) (Figs. 3, 4).

### Clinical features of *FLT3* AML with mutations and fusion genes

In AML patients with *FLT3*-ITD, *NPM1* and *DNMT3A* were the most common mutations. *FLT3*-ITD with both *NPM1* and *DNMT3A* mutations defines a poor prognosis. Three-year OS of *FLT3*-ITD patients with both *NPM1* and *DNMT3A* mutations was 12.7% ± 11.5% (Additional file 2: Fig. S2A), though no significant difference was observed between the four subgroups in *FLT3*-ITD patients in DFS (Additional file 2: Fig. 2B).

**Fig. 5:**
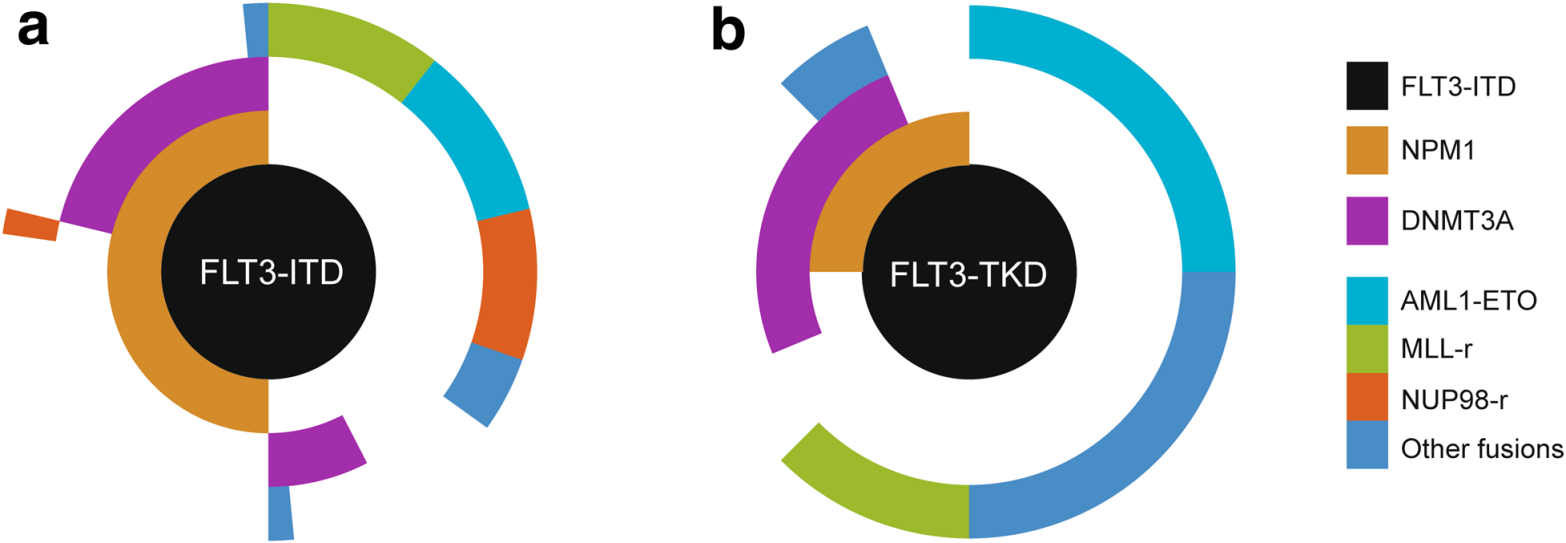
Molecular heterogeneity of AML exemplified by mutational and fusion genes profiling in *FLT3-*AML. Each spoke radiating from the central *FLT3*-ITD or *FLT3*-TKD hub represents the molecular pattern of a single patient. Cooperating mutations are grouped into three tiers according to the function and color-coded according to the figure key, and white space indicates no mutation or fusion. Overall, based on molecular combination, patients are segregated into different subgroups

*NPM1* mutation and fusion genes rarely occurred simultaneously in *FLT3* mutant patients (Figs. 2, 5). Only two patients with *FLT3*-ITD are accompanied by both *NPM1* mutation and fusion genes (*NUP98/HOXA9* and *PRDM16/SKI*, respectively). The *FLT3*-ITD patient with *NUP98/HOXA9* and *NPM1* achieved CR after IA regimen and obtained continuous CR after HSCT. However, the other *FLT3*-ITD patient with *PRDM16/SKI* and *NPM1* was refractory to induction and died of disease progress after four cycles of chemotherapy. The same trend was observed in *FLT3*-TKD group, with only one patient with both *BCR/ABL* and *NPM1* mutation who died during induction due to disease progress. Interestingly, it was also rare to have neither an *NPM1* mutation nor a fusion gene in *FLT3* mutant AML. Only three *FLT3*-ITD patients had neither *NPM1* mutation nor fusion genes, nor did two *FLT3*-TKD AML patients. The outcomes of these five patients were heterogeneous. One patient with *FLT3*-ITD did not receive chemotherapy. The other two patients with *FLT3*-ITD were both refractory to induction. One of the two patients with *FLT3*-TKD without NPM1 or fusions was refractory to induction, and the other one achieved long-term survival after chemotherapy.

**Fig. 6:**
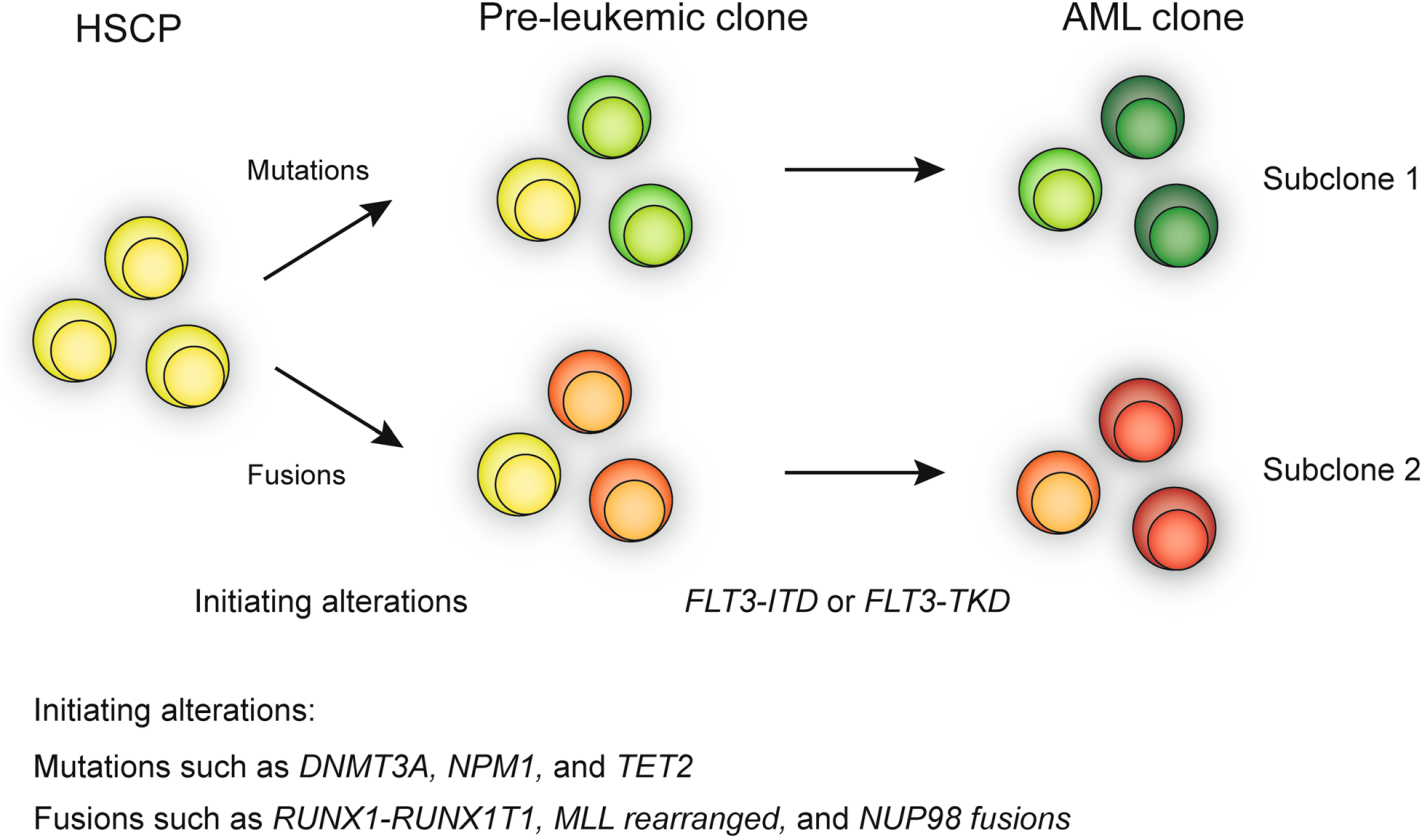
Schematic model for the two paths of evolution of *FLT3* mutant AML. The first step is the occurrence of mutations or fusions, and the second step is the hit of *FLT3*-ITD or *FLT3*-TKD mutations

According to molecular alteration status, *FLT3*-ITD AML patients were divided into *NPM1* + Fusion− group and Fusion + *NPM1*− group. The median age of *NPM1* + Fusion− group was older than that of Fusion + *NPM1*− group, 52 and 36 years old, respectively, *P* = 0.006. *NPM1* mutation was more likely to occur in older patients, while the fusion gene was more likely to be associated with younger age. The average number of mutations in *NPM1* + Fusion− patients was 3.6, while the average number of mutations in Fusion + *NPM1* − group was 2.2, indicating different molecular distributions. *NPM1* mutation was associated with methylation-related mutations such as *DNMT3A*. No difference was observed in the CR rate and survival rate between the two groups (Additional file 3: Fig. S3). Different mutation distributions in *NPM1* + Fusion − group and Fusion + *NPM1* − group could be caused by different pathogenic mechanisms (Table 3; Fig. 6).

**Table 3:** Clinical characteristic and outcomes of patients with NPM1 mutation or fusion genes in *FLT3*-ITD AML

Characteristics	*FLT3*-ITD + NPM1 + Fusion −	*FLT3*-ITD + Fusion + NPM1 −	*P* value^a^
n	31	24	
Age, years Median(range)	52 (14–76)	36 (12–65)	*0.007*
Male n (%)	12 (38.7)	11 (45.8)	0.254
WBC at diagnosis, × 10^9^/L Median(range)	31.4 (1.3–405.1)	10.8 (0.7–306.9)	0.278
Blast in BM, % Median(range)	68.8 (21.2–96.4)	56.4 (11.2–95.6)	0.543
FAB subtype			0.671
M0	0 (0)	0 (0)	
M1	1 (3.3)	1 (4.2)	
M2	8 (26.7)	8 (33.3)	
M4	13 (43.3)	9 (37.5)	
M5	6 (20.0)	5 (20.8)	
M6	0 (0)	1 (4.2)	
Unclassified	0 (0)	0 (0)	
Secondary-AML	2 (6.7)	0 (0)	
Karyotype			0.015
Favorable	0 (0)	7 (29.2)	
Intermediate	27 (87.1)	13 (54.2)	
Normal	22 (71)	9 (37.5)	*0.007*
Others	5 (16.1)	4 (16.7)	
Unfavorable	2 (6.5)	3 (12.5)	
Failed	2 (6.5)	1 (4.2)	
Immunophenotype			
CD34 +	18/25 (72.0)	20/22 (90.9)	0.203
CD13 +	25/26 (96.3)	18/20 (90.0)	0.814
CD33 +	26/26 (100)	21/22 (95.5)	0.458
CD117 +	23/25 (92)	21/22 (95.5)	1.000
CD64 +	10/24 (41.7)	5/11 (45.5)	1.000
Mutations			
Average num	3.6	2.2	*0.000*
* DNMT3A*	13/28 (46.4)	2/22 (9.1)	*0.004*
Methylation-related genes^b^	16/28 (57.1)	4/22 (18.2)	*0.005*
CR, n (%)			
Yes	14/25 (56)	17/24 (70.8)	0.282
Relapse in 1 year			
Yes	16/22 (72.7)	17/20 (85)	0.460

Italic values indicate significance of *P* value (*P* < 0.05)

*WBC* white blood count, *BM* bone marrow, *FAB* French–America–British, *CR* complete remission

^a^P-values for categorical variables are from chi-square test, P-values for continuous variables are from the Mann–Whitney test and Fisher exact test

^b^Methylation-related genes included *DNMT3A*, *IDH1/2*, and *TET2*

## Discussion

*FLT3*-ITD is a late acquired proliferative advantage in leukemogenesis. In addition to *FLT3*-ITD, other molecular alterations are necessary (Fig. 6). Because of the complex pathogenic mechanism, *FLT3*-ITD AML is heterogeneous. *FLT3*-ITD and *FLT3*-TKD mutants show distinct gain-of-function phenotypes with distinct differences in signaling properties and gene expression patterns. Whether *FLT3*-TKD has the same prognostic significance as *FLT3*-ITD is controversial. Comprehensive detection of molecular landscape of *FLT3* mutant AML is significant to establish a risk classification in AML and guide therapy options. Here, we evaluated a cohort of 207 AML patients for mutations in *FLT3* with two targeted sequencing approaches to obtain novel insights into the prognostic relevance of *FLT3* mutations as well as their associations with other molecular markers.

Generally, we observed an overall *FLT3*-ITD and *FLT3*-TKD mutation rate of 28% and 9.7% in 207 de novo AML, consistent with previous reports [21, 22]. Through novel targeted RNA-seq-based NGS, the profile of companying fusion genes with *FLT3* mutations was revealed. The common fusion genes in *FLT3*-ITD had *MLL-*rearrangement and *NUP98*-rearrangement, while the common fusion genes in *FLT3*-TKD had *AML1-ETO* and *MLL*-rearrangement. Two novel fusion genes *PRDM16-SKI* and *EFAN2-ZNF238* were identified in *FLT3*-ITD patients. Gene coexistence analysis revealed unbalanced gene mutation distributions in *FLT3*-ITD, *FLT3*-TKD, and *FLT3*_*wt*_ AML. Gene fusions and *NPM1* mutation are mutually excluded in *FLT3*-ITD and *FLT3*-TKD patients. Their patterns of mutual exclusivity and cooperation among mutated genes suggest that additional driver genetic alterations are required and reveal two evolutionary patterns of *FLT3* pathogenesis. We observed unfavorable impact on CR and survival of *FLT3*-ITD in the whole cohort and NK-AML patients, consistent with other studies [21]. Besides, additional *TET2* mutation further impaired the prognosis of patients with *FLT3*-ITD. In contrast to *FLT3*-ITD mutations, *FLT3*-TKD mutation did not affect the remission rate and survival of AML patients in this study. In patients with *FLT3*-TKD mutations in AML, the main prognostic factor still seems to be the concomitant fusion genes. *MLL* gene rearrangement is an identified adverse prognosis factor. The two patients with *FLT3*-TKD accompanied by *MLL* rearrangement were both primary refractory, while the patients with *AML-ETO* had a relatively good prognosis, 3/4 of them achieving long-term survival. Therefore, attention should be paid to the accompanying molecular abnormalities when stratifying risk in patients with *FLT3*-TKD mutations. *FLT3*-ITD mutation alone is insufficient to drive leukemogenesis, suggesting that additional mutations are necessary for full transformation. Genetic testing incorporating both molecular analysis and cytogenetic karyotyping is an integral part of definition and risk stratification of AML to guide therapy and monitor disease response/relapse [1, 23]. To further understand the pathogenic mechanism and prognostic effect of *FLT3* mutations, gene mutations and gene fusions were examined using two targeted NGS methods in *FLT3*-mutant AML patients. *NPM1* and *DNMT3A* were concomitantly observed together with *FLT3*-ITD. *NPM1* mutation showed a strong correlation with *FLT3*-ITD in previous reports [8, 24, 25]. *NPM1* is considered as one of the early cooperating mutations in leukemia leukemogenesis (Fig. 6) [26]. *DNMT3A* mutation is another common mutation in patients with *FLT3*-ITD. Epigenetics plays an important role in leukemogenesis. In Jifeng Yu et al*.*’s recent study, older AML patients (≥ 60 years) showed association with more incidence of DNA methylation compared with younger AML patients (87.7% vs. 75.4%, *P* = 0.0425) [27]. D*NMT3A* mutation functions, as an epigenetic regulator, are associated with aging [28]. This could explain the elder distributions of age of *NPM1* mutation subgroup. We found that patients with comutation of *FLT3*-ITD and *TET2* mutation had shorter survival compared to patients with *FLT3*-ITD mutation and wildtype *TET2*, identifying *FLT3-*ITD*/TET2* bimutation as a high-risk AML subgroup. *TET2* mutation and *FLT3*-ITD cooperatively remodeled DNA methylation and gene expression and triggered AML in vivo. Besides, the induced AML cell demonstrated refractory to standard AML chemotherapy and *FLT3* targeted treatment [29]. We previously reported that *TET2* mutation is an unfavorable prognostic factor in AML patients [30]. Furthermore, *TET2* mutation with *FLT3*-ITD could further stratify AML patients with intermediate-risk cytogenetics. Interestingly, the mutations in *Ra*s, *CEBPA*, and *TP53* were found to be excluded in *FLT3* mutant AML. *CEBPA* mutation was reported to be restricted in normal karyotype without *FLT3*-ITD and *NPM1* mutation [31]. *Ras* also led to secondary events that occur later during leukemogenesis. Similar to *Stirewalt*’s study, the same negative association was observed between *Ras* mutation and *FLT3* mutations in our study [32]. Most *TP53* mutation is associated with abnormal cytogenetics, especially abnormalities in chromosomes 5 and 7, while *FLT3*-ITD is associated with normal karyotype [32]. These exclusive relationship between *FLT3* mutation and mutations in *Ra*s, *CEBPA*, and *TP53* probably indicate that the use of a differential detection panel in genetic mutations may be convenient and economical [33].

The fusion genes are important pathogenic mechanism of leukemogenesis. It is considered as a potential therapeutic target and MRD monitoring marker. We found that *MLL-*rearrangement and *NUP98-*rearrangement are both recurrent fusion genes in *FLT3*-ITD, and their partner genes are multitudinous. In a Genome Atlas Research, 118 gene fusions were found in 178 de novo AML samples, including 74 reported recurring events and 57 novel gene fusions; most of them were not detected using cytogenetic studies [24]. The karyotype analysis relies on experts’ experience, and it is likely to be missed if the changes in chromosomal appearance after translocation are not easily discernible. Besides, the karyotype results may be inconsistent with the fusion gene expression under some conditions. In our study, 32 *FLT3*-mutant patients were identified with fusions, 28 of which were generated by translocations. However, only seven fusions showed consistent karyotype results.

PCR detection relies on targeted primer design; novel/rare fusion genes and common genes fusing at a rare site could be missed. On the other hand, even though chimeric RNA is almost the product of chromosomal rearrangement at the DNA level, it can also be generated from *trans*-splicing and *cis*-splicing between neighboring genes in some cases, which is only detectable at the RNA level than the DNA level [34–36]. Thus, RNA-based fusion gene detection is more comprehensive. The advantage of NGS is that it can detect atypical sites of classical fusions, identify fusions involving multiple fusion partner genes, and discover rare and unknown fusions [37]. In this study, 21 of 52 patients with *FLT3*-ITD had fusion genes detected by NGS, and the incidence of fusion genes is 40.4%. The most common fusions in *FLT3*-ITD included *MLL*-rearranged and *NUP98*-rearranged. The *MLL* fusion was associated with the fewest number of mutant genes in the newly diagnosed AML, indicating that the *MLL* gene alterations are very strong AML-initiating factors. Besides, *NPM1* and *DNMT3A* gene mutations were exclusive in *MLL* fusions [24, 38]. *MLL* rearrangement accounts for about 10% of AML, and the prognosis is very poor. The median age of onset of leukemia in infants and young children closely related to *MLL* rearrangement is only six months, suggesting that *MLL* rearrangement is a very powerful pathogenic factor. A high expression of *FLT3* is frequently observed in *MLL-*rearranged AML, but in vivo experiments showed that could induce AML independent of the *FLT3* signaling pathway [39, 40]. A long-distance inverse PCR can be used to characterize *MLL* rearrangement; identification and distribution of *MLL* rearrangements of 579 AML samples including infants, pediatric, and adults were studied [41]. The most frequent fusion genes were *MLL-MLLT3/AF9* (28.8%), *MLL-MLLT10/AF10* (15.2%), *MLL-ELL* (11.4%), *MLL*-PTDs (11.4%), *MLL-MLLT4/AF6* (9.5%), *MLL-MLLT1/ENL* (4.0%), *MLL-SEPT6* (1.9%), and *MLL-MLLT6/AF17* (1.6%). Adult AML patients were characterized by a higher frequency of *MLL*-PTD at 23.4% (64/272) compared to none and 1.9% in infants and pediatric, respectively. In this study, *MLL*-PTD was positive in four patients with *FLT3*-ITD, which is the second most frequent fusion in *FLT3*-ITD in our study. *MLL*-PTD is not sufficient to cause leukemia alone; an additional *FLT3*-ITD could trigger leukemia in mice [42]. Sun et al. identified the most frequent mutation in *MLL*-PTD, which was *FLT3*, and *NPM1* was mutually exclusive with *MLL*-PTD, exhibiting the same trend as in this study [26]. Interestingly, *NPM1* was wild type in these four *MLL*-PTD patients. *MLL*-PTD functioned as an early clonal driver mutation, while *FLT3*-ITD was acquired later.

*NUP98-NSD1*, created by the translocation of juxtaposition of Nucleoporin 98 (*NUP98*) and nuclear receptor binding SET-domain Protein 1 (*NSD1*) gene, is a common type of translocation in *FLT3*-ITD AML patients [10]. The incidence of translocation involving *NUP98* in AML is very low, only 3% in adult AML [43]. It is reported that the frequency of *NUP98-NSD1* in *FLT3*-ITD can reach 15%, and the frequency of *NUP98-NSD1* combined with *FLT3*-ITD is 82% [44]. The incidence of *NUP98* fusion accounted for 33.3% fusion genes in *FLT3*-ITD, and the incidence of *NUP98-NSD1* in *FLT3*-ITD was 6.7% in our study, reflecting a strong synergistic effect. The lower incidence of *NUP98-NSD1* in *FLT3*-ITD compared to the previous report is probably due to the insufficient number of cases. When *NUP98*-*NSD1* and *FLT3*-ITD occur simultaneously, the CR rate is less than 30% in AML patients with concurrent *NUP98*-*NSD1* and *FLT3*-ITD, and the patient’s prognosis is extremely poor [45].

The *FLT3*-TKD is positively correlated with normal karyotype, and the incidence of *FLT3*-TKD is 5–10% in normal karyotype AML [22, 46]. In the largest clinical study on *FLT3*-TKD, *FLT3*-TKD mutation alone did not affect prognosis [22]. Though no difference was observed in the incidence of *NPM1* mutation in patients with *FLT3*-TKD and *FLT3*wt, *NPM1* was observed to be one of the most common mutations in *FLT3*-TKD in this study. Expression of *FLT3*-TKD is insufficient to trigger leukemia in mice; however, a co-*NPM1* mutation actively led to the onset of AML in mice. *NPM1*c altered the cellular localization of *FLT3*-TKD, leading to the aberrant activation of downstream STAT5 signaling pathway [47]. Interestingly, patients with *FLT3*-TKD and *NPM1* comutation had a better prognosis than patients with *FLT3*-TKD or *NPM1* mutation alone [22, 48]. *FLT3*-TKD had an unfavorable influence on prognosis in t(15;17)/*PML-RARA* and *MLL*-PTD/TKD double-mutated cases. Compared with *FLT3*-ITD, *FLT3*-TKD exhibits different molecular genetic profiles. The most frequent fusion gene in *FLT3*-TKD group was *AML1-ETO*. The high correlation between *FLT3*-TKD and *AML1-ETO* is probably one of the reasons why it has no adverse effect on prognosis. *AML1-ETO* fusion is one of the most common fusions in AML. In *AML1-ETO* AML patients, combined gene mutations are most frequently involved in the signal transduction pathway, including *FLT3*, *KIT*, and *NRAS* [49, 50]. The incidence of *FLT3* mutation in core-binding factor (CBF) AML is 5–10%. *FLT3* mutation combined with *AML1-ETO* gene fusion can lead to the onset of leukemia [9, 51]. In our previous study, 21 patients with *AML1-ETO* fusion-positive AML had a higher relapse rate and mortality with an *FLT3* gene expression greater than 35% [52]. *FLT3*-ITD attenuates the good prognosis of *AML1-ETO* to some extent.

Because *FLT3* mutation is insufficient to induce leukemia, additional gene aberration is necessary. We comprehensively examined the molecular genetic background of *FLT3* mutant AML using two second-generation sequencing methods. In previous studies, *FLT3* was found to be a late acquired genetic change; our results revealed two molecular collaborative patterns of *FLT3* mutation in leukemia progression. Initiation of molecular alterations includes mutations and fusions. Initiation of cooperative gene mutation mainly includes *NPM1* mutation and methylation-modified genes such as *DNMT3A* and *TET2*. In the other *FLT3* mutant patients, fusions play an important role in leukemogenesis, especially the *MLL*-rearranged, *NUP98* fusions, and *AML1-ETO* (Fig. 6).

## Conclusions

In summary, this study elucidated the coexisting molecular landscape of AML with *FLT3*-ITD and *FLT3*-TKD mutations by NGS, revealing two patterns of two paths of evolution of *FLT3* mutant AML. We confirmed the unfavorable prognostic effect of *FLT3*-ITD and no influence of *FLT3*-TKD on prognosis. Patients with *FLT3*-ITD/*TET2* bimutation are a high-risk subgroup. Finally, this study provides further insight into the role that genetic alterations including fusion genes and mutations may eventually lead to the development of effective and precise targeted therapy in *FLT3* mutated AML.

## Supplementary Information

**Additional file 1: Figure S1.** Treatment flow diagram.

**Additional file 2: Figure S2.** Overall survival(A) and Disease-Free Survival(B) curves of *FLT3*-ITD patients divided into four subgroups according to *NPM1* and *DNMT3A* status. The subscript wt and mut represents wildtype and mutant.

**Additional file 3: Figure S3**. Overall survival(A) and Disease-Free Survival(B) curves of *FLT3*-ITD AML patients with *NPM1* mutation or fusion genes.

**Additional file 4: Table S1**. Gene penal list by next-generation sequencing.

## Data Availability

The datasets generated and/or analyzed during this study are not publicly available due to privacy policy but are available from the corresponding author on reasonable request.

## Abbreviations

ITD: Internal tandem duplications
TKD: Tyrosine kinase domain
AML: Acute myeloid leukemia
NGS: Next-generation sequencing
WBC: White blood count
NK: Normal karyotype
CR: Complete remission
OS: Overall survival
DFS: Disease-free survival
EFS: Event-free survival
CML: Chronic myelogenous leukemia
MRD: Minimal residual disease
