# Supplementary figures and images for: Profiling of somatic mutations and fusion genes in acute myeloid leukemia patients with FLT3-ITD or FLT3-TKD mutation at diagnosis reveals distinct evolutionary patterns
Source: Exp Hematol Oncol. 2021 Apr 9;10:27. doi: 10.1186/s40164-021-00207-4 (PMC8033687; doi:10.1186/s40164-021-00207-4)

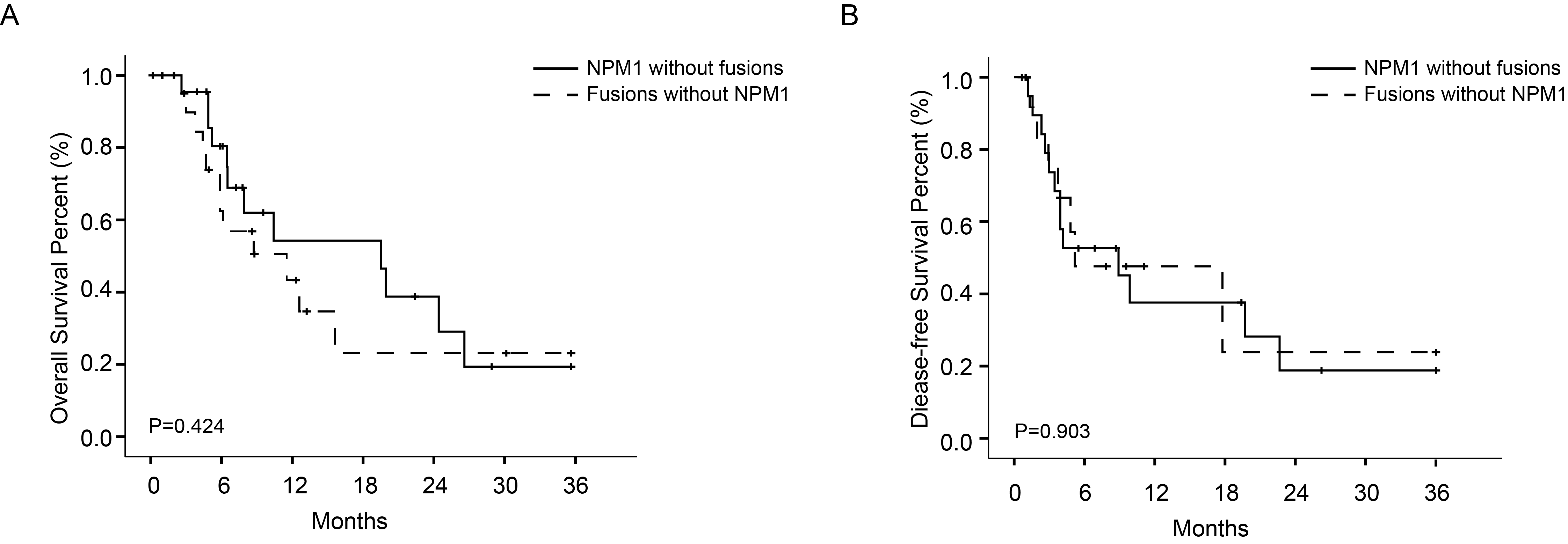

Supplement: Supplementary file 1 — Additional file 1: Figure S1. Treatment flow diagram. [file 40164_2021_207_MOESM1_ESM.tif]

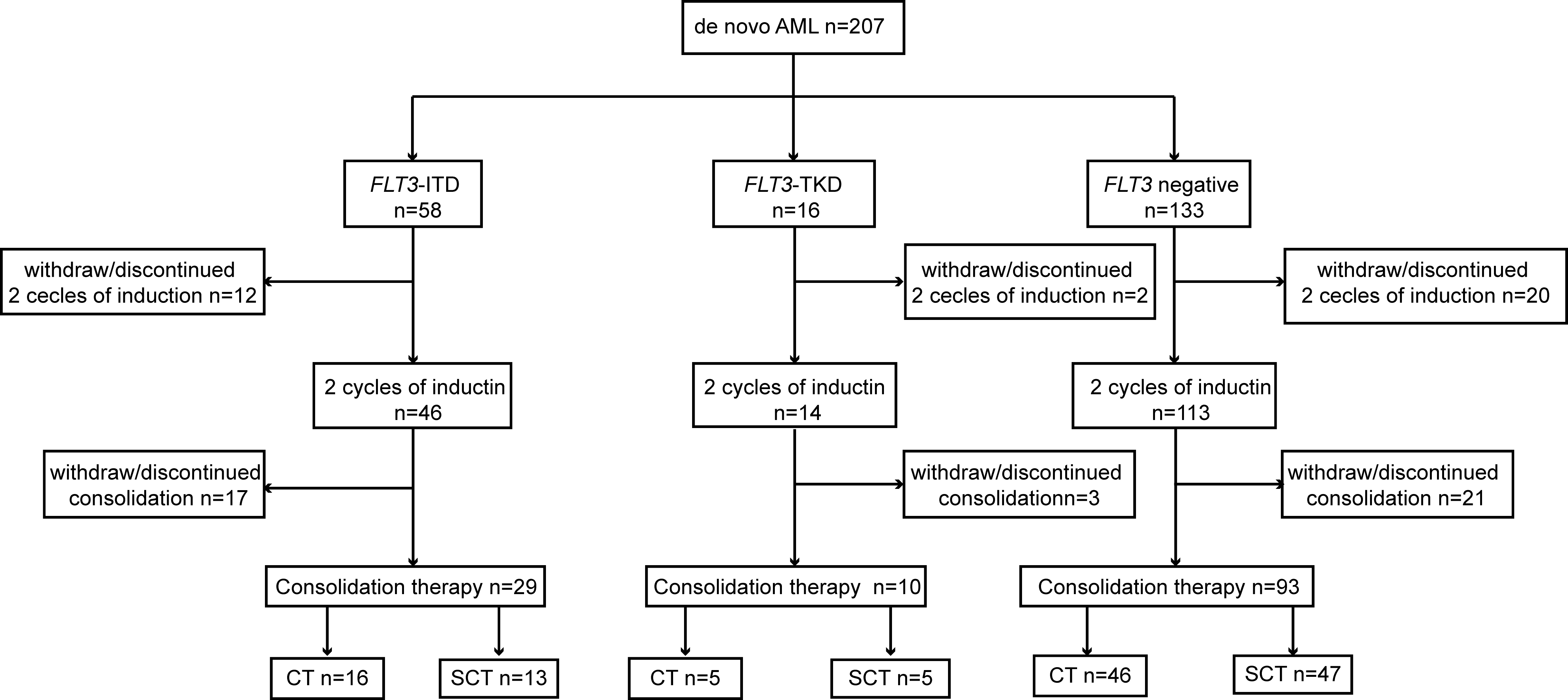

Supplement: Supplementary file 2 — Additional file 2: Figure S2. Overall survival(A) and Disease-Free Survival(B) curves of FLT3-ITD patients divided into four subgroups according to NPM1 and DNMT3A status. The subscript wt and mut represents wildtype and mutant. [file 40164_2021_207_MOESM2_ESM.tif]

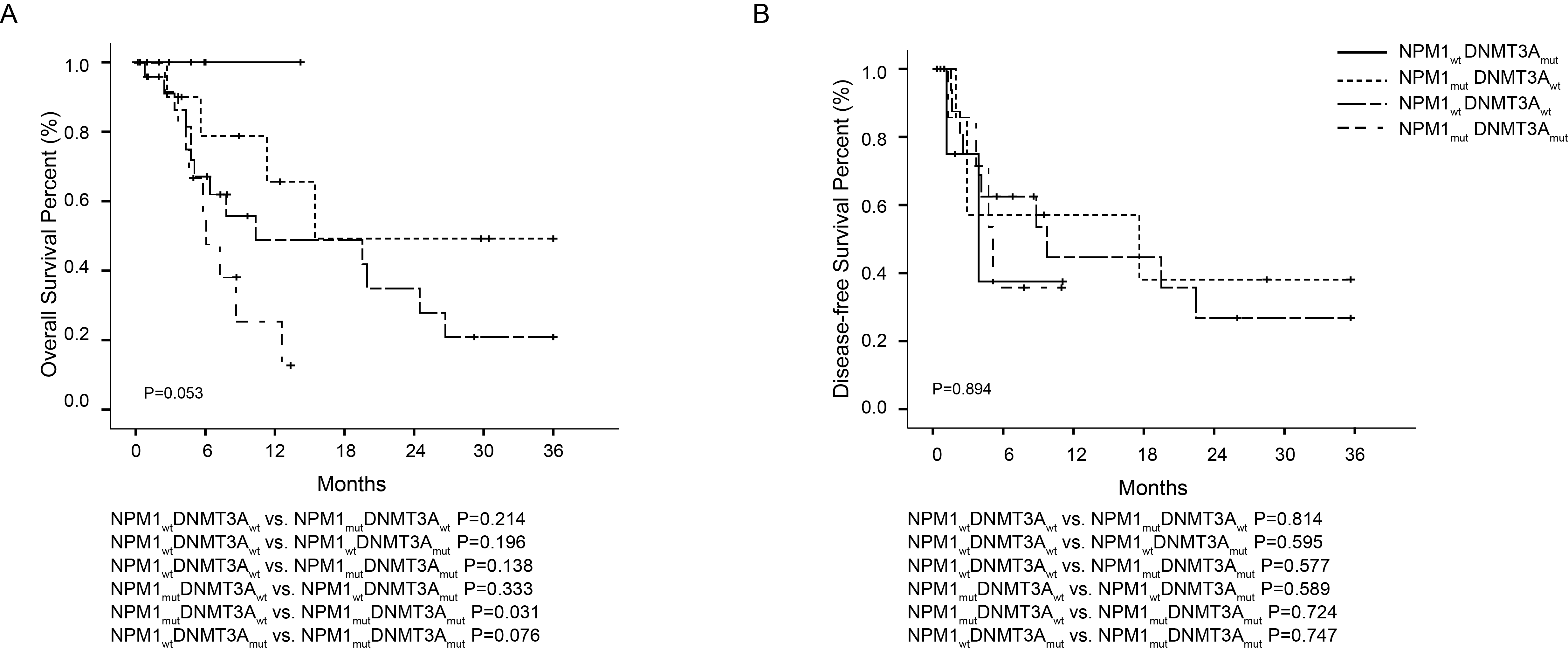

Supplement: Supplementary file 3 — Additional file 3: Figure S3. Overall survival(A) and Disease-Free Survival(B) curves of FLT3-ITD AML patients with NPM1 mutation or fusion genes. [file 40164_2021_207_MOESM3_ESM.tif]
