# Supplementary material for: Profiling of somatic mutations and fusion genes in acute myeloid leukemia patients with FLT3-ITD or FLT3-TKD mutation at diagnosis reveals distinct evolutionary patterns
Source: Exp Hematol Oncol. 2021 Apr 9;10:27. doi: 10.1186/s40164-021-00207-4 (PMC8033687; doi:10.1186/s40164-021-00207-4)
Supplement: Supplementary file 4 — Additional file 4: Table S1. Gene penal list by next-generation sequencing. [file 40164_2021_207_MOESM4_ESM.docx]

Table S1. Gene penal list by next generation sequencing.

| Gene name | Other gene names | Gene ID | Location | Number of fusions |
| --- | --- | --- | --- | --- |
| KMT2A | ALL-1\|CXXC7\|HRX\|HTRX1\|MLL\|MLL-AF9\|MLL/GAS7\|MLL1\|MLL1A\|TET1-MLL\|TRX1\|WDSTS | 4297 | 11q23 | 66 |
| ETV6 | TEL\|TEL/ABL\|THC5 | 2120 | 12p13 | 41 |
| ALK | CD246\|NBLST3 | 238 | 2p23 | 35 |
| RARA | NR1B1\|RAR | 5914 | 17q21 | 34 |
| RUNX1 | AML1\|AML1-EVI-1\|AMLCR1\|CBF2alpha\|CBFA2\|EVI-1\|PEBP2aB\|PEBP2alpha | 861 | 21q22.3 | 34 |
| NUP98 | ADIR2\|NUP196\|NUP96 | 4928 | 11p15.5 | 33 |
| BCL6 | BCL5\|BCL6A\|LAZ3\|ZBTB27\|ZNF51 | 604 | 3q27 | 31 |
| PDGFRB | CD140B\|IBGC4\|IMF1\|JTK12\|KOGS\|PDGFR\|PDGFR-1\|PDGFR1\|PENTT | 5159 | 5q33.1 | 28 |
| EWSR1 | EWS\|EWS-FLI1\|bK984G1.4 | 2130 | 22q12.2 | 27 |
| FOXP1 | 12CC4\|HSPC215\|MFH\|QRF1\|hFKH1B | 27086 | 3p14.1 | 27 |
| FGFR1 | BFGFR\|CD331\|CEK\|FGFBR\|FGFR-1\|FLG\|FLT-2\|FLT2\|HBGFR\|HH2\|HRTFDS\|KAL2\|N-SAM\|OGD\|bFGF-R-1 | 2260 | 8p11.23-p11.22 | 22 |
| ROS1 | MCF3\|ROS\|c-ros-1 | 6098 | 6q22 | 22 |
| PAX5 | ALL3\|BSAP | 5079 | 9p13 | 22 |
| RET | CDHF12\|CDHR16\|HSCR1\|MEN2A\|MEN2B\|MTC1\|PTC\|RET-ELE1\|RET51 | 5979 | 10q11.2 | 21 |
| JAK2 | JTK10\|THCYT3 | 3717 | 9p24 | 18 |
| MYC | MRTL\|MYCC\|bHLHe39\|c-Myc | 4609 | 8q24.21 | 17 |
| HMGA2 | BABL\|HMGI-C\|HMGIC\|LIPO\|STQTL9 | 8091 | 12q15 | 17 |
| ABL1 | ABL\|JTK7\|bcr/abl\|c-ABL\|c-ABL1\|p150\|v-abl | 25 | 9q34.1 | 16 |
| LPP | - | 4026 | 3q28 | 16 |
| ERG | erg-3\|p55 | 2078 | 21q22.3 | 14 |
| KAT6A | MOZ\|MRD32\|MYST-3\|MYST3\|RUNXBP2\|ZC2HC6A\|ZNF220 | 7994 | 8p11 | 14 |
| MLLT10 | AF10 | 8028 | 10p12 | 13 |
| ETV1 | ER81 | 2115 | 7p21.3 | 13 |
| FUS | ALS6\|ETM4\|FUS1\|HNRNPP2\|POMP75\|TLS | 2521 | 16p11.2 | 12 |
| PBX1 | - | 5087 | 1q23 | 11 |
| MYB | Cmyb\|c-myb\|c-myb_CDS\|efg | 4602 | 6q22-q23 | 11 |
| PLAG1 | PSA\|SGPA\|ZNF912 | 5324 | 8q12 | 11 |
| ETV4 | E1A-F\|E1AF\|PEA3\|PEAS3 | 2118 | 17q21.31 | 11 |
| BCR | ALL\|BCR1\|CML\|D22S11\|D22S662\|PHL | 613 | 22q11.23 | 10 |
| MECOM | AML1-EVI-1\|EVI1\|MDS1\|MDS1-EVI1\|PRDM3 | 2122 | 3q26.2 | 10 |
| NUP214 | CAIN\|CAN\|D9S46E\|N214\|p250 | 8021 | 9q34.1 | 10 |
| TFE3 | RCCP2\|RCCX1\|TFEA\|bHLHe33 | 7030 | Xp11.22 | 10 |
| CREBBP | CBP\|KAT3A\|RSTS | 1387 | 16p13.3 | 10 |
| CCDC6 | D10S170\|H4\|PTC\|TPC\|TST1 | 8030 | 10q21 | 10 |
| CLTC | CHC\|CHC17\|CLH-17\|CLTCL2\|Hc | 1213 | 17q23.1 | 10 |
| COL1A1 | EDSC\|OI1\|OI2\|OI3\|OI4 | 1277 | 17q21.33 | 9 |
| ACTB | BRWS1\|PS1TP5BP1 | 60 | 7p22 | 9 |
| BCL2 | Bcl-2\|PPP1R50 | 596 | 18q21.3 | 8 |
| FGFR3 | ACH\|CD333\|CEK2\|HSFGFR3EX\|JTK4 | 2261 | 4p16.3 | 8 |
| TCF3 | E2A\|E47\|ITF1\|TCF-3\|VDIR\|bHLHb21 | 6929 | 19p13.3 | 8 |
| MAML2 | MAM-3\|MAM2\|MAM3\|MLL-MAML2 | 84441 | 11q21 | 8 |
| TMPRSS2 | PP9284\|PRSS10 | 7113 | 21q22.3 | 8 |
| AFF3 | LAF4\|MLLT2-like | 3899 | 2q11.2-q12 | 8 |
| CCND1 | BCL1\|D11S287E\|PRAD1\|U21B31 | 595 | 11q13 | 7 |
| JAZF1 | TIP27\|ZNF802 | 221895 | 7p15.2-p15.1 | 7 |
| CCND2 | KIAK0002\|MPPH3 | 894 | 12p13 | 7 |
| BCL11B | ATL1\|ATL1-alpha\|ATL1-beta\|ATL1-delta\|ATL1-gamma\|CTIP-2\|CTIP2\|RIT1\|ZNF856B\|hRIT1-alpha | 64919 | 14q32.2 | 7 |
| TPM3 | CAPM1\|CFTD\|HEL-189\|HEL-S-82p\|NEM1\|OK/SW-cl.5\|TM-5\|TM3\|TM30\|TM30nm\|TM5\|TPMsk3\|TRK\|hscp30 | 7170 | 1q21.2 | 7 |
| NPM1 | B23\|NPM | 4869 | 5q35.1 | 6 |
| PDGFRA | CD140A\|PDGFR-2\|PDGFR2\|RHEPDGFRA | 5156 | 4q12 | 6 |
| PICALM | CALM\|CLTH\|LAP | 8301 | 11q14 | 6 |
| SS18 | SSXT\|SYT | 6760 | 18q11.2 | 6 |
| TAL1 | SCL\|TCL5\|bHLHa17\|tal-1 | 6886 | 1p32 | 6 |
| AKT3 | MPPH\|MPPH2\|PKB-GAMMA\|PKBG\|PRKBG\|RAC-PK-gamma\|RAC-gamma\|STK-2 | 10000 | 1q44 | 6 |
| USP6 | HRP1\|TRE17\|TRE2\|TRESMCR\|Tre-2\|USP6-short | 9098 | 17p13 | 6 |
| TFG | HMSNP\|SPG57\|TF6\|TRKT3 | 10342 | 3q12.2 | 6 |
| SLC45A3 | IPCA-2\|IPCA-6\|IPCA-8\|IPCA6\|PCANAP2\|PCANAP6\|PCANAP8\|PRST | 85414 | 1q32.1 | 6 |
| TAF15 | Npl3\|RBP56\|TAF2N\|TAFII68 | 8148 | 17q11.1-q11.2 | 5 |
| PAX3 | CDHS\|HUP2\|WS1\|WS3 | 5077 | 2q35 | 5 |
| TRIM24 | PTC6\|RNF82\|TF1A\|TIF1\|TIF1A\|TIF1ALPHA\|hTIF1 | 8805 | 7q32-q34 | 5 |
| CDK6 | MCPH12\|PLSTIRE | 1021 | 7q21-q22 | 5 |
| PRDM16 | CMD1LL\|LVNC8\|MEL1\|PFM13 | 63976 | 1p36.23-p33 | 5 |
| CBFA2T3 | ETO2\|MTG16\|MTGR2\|ZMYND4 | 863 | 16q24 | 4 |
| PRKAR1A | ACRDYS1\|ADOHR\|CAR\|CNC\|CNC1\|PKR1\|PPNAD1\|PRKAR1\|TSE1 | 5573 | 17q24.2 | 4 |
| TLX3 | HOX11L2\|RNX | 30012 | 5q35.1 | 4 |
| BCL3 | BCL4\|D19S37 | 602 | 19q13.1-q13.2 | 4 |
| DDIT3 | CEBPZ\|CHOP\|CHOP-10\|CHOP10\|GADD153 | 1649 | 12q13.1-q13.2 | 3 |
| FOXO4 | AFX\|AFX1\|MLLT7 | 4303 | Xq13.1 | 3 |
| HOXA9 | ABD-B\|HOX1\|HOX1.7\|HOX1G | 3205 | 7p15.2 | 3 |
| MALT1 | IMD12\|MLT\|MLT1 | 10892 | 18q21 | 3 |
| NKX2-5 | CHNG5\|CSX\|CSX1\|HLHS2\|NKX2.5\|NKX2E\|NKX4-1\|VSD3 | 1482 | 5q34 | 3 |
| PML | MYL\|PP8675\|RNF71\|TRIM19 | 5371 | 15q22 | 3 |
| RPN1 | OST1\|RBPH1 | 6184 | 3q21.3 | 3 |
| CCND3 | - | 896 | 6p21 | 3 |
| RHOH | ARHH\|TTF | 399 | 4p13 | 3 |
| FOXO1 | FKH1\|FKHR\|FOXO1A | 2308 | 13q14.1 | 3 |
| NTRK1 | MTC\|TRK\|TRK1\|TRKA\|Trk-A\|p140-TrkA | 4914 | 1q21-q22 | 3 |
| TBL1XR1 | C21\|DC42\|IRA1\|TBLR1 | 79718 | 3q26.32 | 3 |
| EBF1 | COE1\|EBF\|O/E-1\|OLF1 | 1879 | 5q34 | 3 |
| AFF1 | AF4\|MLLT2\|PBM1 | 4299 | 4q21 | 2 |
| BIRC3 | AIP1\|API2\|CIAP2\|HAIP1\|HIAP1\|MALT2\|MIHC\|RNF49\|c-IAP2 | 330 | 11q22 | 2 |
| CBFB | PEBP2B | 865 | 16q22.1 | 2 |
| FIP1L1 | FIP1\|Rhe\|hFip1 | 81608 | 4q12 | 2 |
| HFE2 | HFE2A\|HJV\|JH\|RGMC | 148738 | 1q21.1 | 2 |
| HIP1 | HIP-I\|ILWEQ\|SHON\|SHONbeta\|SHONgamma | 3092 | 7q11.23 | 2 |
| HOXA11 | HOX1\|HOX1I | 3207 | 7p15.2 | 2 |
| MAF | AYGRP\|CCA4\|CTRCT21\|c-MAF | 4094 | 16q22-q23 | 2 |
| MYO18A | MYSPDZ\|SPR210 | 399687 | 17q11.2 | 2 |
| SET | 2PP2A\|I2PP2A\|IGAAD\|IPP2A2\|PHAPII\|TAF-I\|TAF-IBETA | 6418 | 9q34 | 2 |
| TLX1 | HOX11\|TCL3 | 3195 | 10q24 | 2 |
| CRLF2 | CRL2\|CRLF2Y\|TSLPR | 64109 | Xp22.3; Yp11.3 | 2 |
| BCL9 | LGS | 607 | 1q21 | 2 |
| LMO1 | RBTN1\|RHOM1\|TTG1 | 4004 | 11p15 | 2 |
| IRF4 | LSIRF\|MUM1\|NF-EM5\|SHEP8 | 3662 | 6p25.3 | 2 |
| BCL10 | CARMEN\|CIPER\|CLAP\|IMD37\|c-E10\|mE10 | 8915 | 1p22 | 2 |
| RANBP17 | - | 64901 | 5q34 | 2 |
| KDM4C | GASC1\|JHDM3C\|JMJD2C\|TDRD14C | 23081 | 9p24.1 | 2 |
| PDCD1LG2 | B7DC\|Btdc\|CD273\|PD-L2\|PDCD1L2\|PDL2\|bA574F11.2 | 80380 | 9p24.2 | 2 |
| RABEP1 | RAB5EP\|RABPT5 | 9135 | 17p13.2 | 2 |
| CIC | - | 23152 | 19q13.2 | 2 |
| NOTCH1 | AOS5\|AOVD1\|TAN1\|hN1 | 4851 | 9q34.3 | 2 |
| ERVW-1 | ENV\|ENVW\|ERVWE1\|HERV-7q\|HERV-W-ENV\|HERV7Q\|HERVW\|HERVWENV | 30816 | 7q21.2 | 2 |
| SSX1 | CT5.1\|SSRC | 6756 | Xp11.23 | 2 |
| A2M | A2MD\|CPAMD5\|FWP007\|S863-7 | 2 | 12p13.31 | 1 |
| ABI1 | ABI-1\|ABLBP4\|E3B1\|NAP1BP\|SSH3BP\|SSH3BP1 | 10006 | 10p11.2 | 1 |
| ABL2 | ABLL\|ARG | 27 | 1q25.2 | 1 |
| ACTN4 | ACTININ-4\|FSGS\|FSGS1 | 81 | 19q13 | 1 |
| BCOR | ANOP2\|MAA2\|MCOPS2 | 54880 | Xp11.4 | 1 |
| BIN2 | BRAP-1 | 51411 | 12q13 | 1 |
| CBFA2T2 | EHT\|MTGR1\|ZMYND3\|p85 | 9139 | 20q11 | 1 |
| DDX10 | HRH-J8 | 1662 | 11q22-q23 | 1 |
| DEK | D6S231E | 7913 | 6p22.3 | 1 |
| ELL | C19orf17\|ELL1\|MEN\|PPP1R68 | 8178 | 19p13.1 | 1 |
| EPS15 | AF-1P\|AF1P\|MLLT5 | 2060 | 1p32 | 1 |
| FOXO3 | AF6q21\|FKHRL1\|FKHRL1P2\|FOXO2\|FOXO3A | 2309 | 6q21 | 1 |
| GIT2 | CAT-2\|CAT2 | 9815 | 12q24.1 | 1 |
| HLF | - | 3131 | 17q22 | 1 |
| HOXA13 | HOX1\|HOX1J | 3209 | 7p15.2 | 1 |
| HOXC11 | HOX3H | 3227 | 12q13.3 | 1 |
| HOXD13 | BDE\|BDSD\|HOX4I\|SPD | 3239 | 2q31.1 | 1 |
| IL3 | IL-3\|MCGF\|MULTI-CSF | 3562 | 5q31.1 | 1 |
| MAFB | KRML\|MCTO | 9935 | 20q12 | 1 |
| MKL1 | BSAC\|MAL\|MRTF-A | 57591 | 22q13 | 1 |
| MLF1 | - | 4291 | 3q25.1 | 1 |
| MLLT1 | ENL\|LTG19\|YEATS1 | 4298 | 19p13.3 | 1 |
| MLLT11 | AF1Q | 10962 | 1q21 | 1 |
| MLLT3 | AF9\|YEATS3 | 4300 | 9p22 | 1 |
| MLLT4 | AF6\|MLL-AF6 | 4301 | 6q27 | 1 |
| MLLT6 | AF17 | 4302 | 17q21 | 1 |
| MYH11 | AAT4\|FAA4\|SMHC\|SMMHC | 4629 | 16p13.11 | 1 |
| NUMA1 | NMP-22\|NUMA | 4926 | 11q13 | 1 |
| PDGFB | IBGC5\|PDGF-2\|PDGF2\|SIS\|SSV\|c-sis | 5155 | 22q13.1 | 1 |
| PRRX1 | AGOTC\|PHOX1\|PMX1\|PRX-1\|PRX1 | 5396 | 1q24 | 1 |
| RBM15 | OTT\|OTT1\|SPEN | 64783 | 1p13 | 1 |
| RUNX1T1 | AML1-MTG8\|AML1T1\|CBFA2T1\|CDR\|ETO\|MTG8\|ZMYND2 | 862 | 8q22 | 1 |
| 6-Sep | SEP2\|SEPT2 | 23157 | Xq24 | 1 |
| SSX2 | CT5.2\|CT5.2A\|HD21\|HOM-MEL-40\|SSX | 6757 | Xp11.22 | 1 |
| STAT5B | STAT5 | 6777 | 17q11.2 | 1 |
| STIL | MCPH7\|SIL | 6491 | 1p32 | 1 |
| ZBTB16 | PLZF\|ZNF145 | 7704 | 11q23.1 | 1 |
| ZMYM2 | FIM\|MYM\|RAMP\|SCLL\|ZNF198 | 7750 | 13q11-q12 | 1 |
| MYCN | MODED\|N-myc\|NMYC\|ODED\|bHLHe37 | 4613 | 2p24.3 | 1 |
| TERT | CMM9\|DKCA2\|DKCB4\|EST2\|PFBMFT1\|TCS1\|TP2\|TRT\|hEST2\|hTRT | 7015 | 5p15.33 | 1 |
| NFKB2 | CVID10\|H2TF1\|LYT-10\|LYT10\|NF-kB2\|p100\|p52 | 4791 | 10q24 | 1 |
| CEBPG | GPE1BP\|IG/EBP-1 | 1054 | 19q13.11 | 1 |
| LHX2 | LH2\|hLhx2 | 9355 | 9q33.3 | 1 |
| TPD52 | D52\|N8L\|PC-1\|PrLZ\|hD52 | 7163 | 8q21.13 | 1 |
| ABCA13 | - | 154664 | 7p12.3 | 1 |
| LCK | IMD22\|LSK\|YT16\|p56lck\|pp58lck | 3932 | 1p34.3 | 1 |
| GPR34 | LYPSR1 | 2857 | Xp11.4 | 1 |
| IRF8 | H-ICSBP\|ICSBP\|ICSBP1\|IMD32A\|  IMD32B\|IRF-8 | 3394 | 16q24.1 | 1 |
| BANK1 | BANK | 55024 | 4q24 | 1 |
| TNFSF13 | APRIL\|CD256\|TALL-2\|TALL2\|TRDL-1\|UNQ383/PRO715\|ZTNF2 | 8741 | 17p13.1 | 1 |
| IRS4 | IRS-4\|PY160 | 8471 | Xq22.3 | 1 |
| DDX6 | HLR2\|P54\|RCK | 1656 | 11q23.3 | 1 |
| CEBPA | C/EBP-alpha\|CEBP | 1050 | 19q13.1 | 1 |
| GRIP1 | GRIP | 23426 | 12q14.3 | 1 |
| LMO2 | RBTN2\|RBTNL1\|RHOM2\|TTG2 | 4005 | 11p13 | 1 |
| LYL1 | bHLHa18 | 4066 | 19p13.2 | 1 |
| PPP1CB | HEL-S-80p\|PP-1B\|PP1B\|PP1beta\|PPP1CD | 5500 | 2p23 | 1 |
| EGFR | ERBB\|ERBB1\|HER1\|NISBD2\|PIG61\|mENA | 1956 | 7p12 | 1 |
| BMI1 | FLVI2/BMI1\|PCGF4\|RNF51\|flvi-2/bmi-1 | 648 | 10p11.23 | 1 |
| FCRL4 | CD307d\|FCRH4\|IGFP2\|IRTA1 | 83417 | 1q21 | 1 |
| BACH2 | BTBD25 | 60468 | 6q15 | 1 |
| KDSR | DHSR\|FVT1\|SDR35C1 | 2531 | 18q21.3 | 1 |
| IGF2BP1 | CRD-BP\|CRDBP\|IMP-1\|IMP1\|VICKZ1\|ZBP1 | 10642 | 17q21.32 | 1 |
| DUSP22 | JKAP\|JSP-1\|JSP1\|LMW-DSP2\|LMWDSP2\|MKP-x\|MKPX\|VHX | 56940 | 6p25.3 | 1 |
| BCL11A | BCL11A-L\|BCL11A-S\|BCL11A-XL\|BCL11a-M\|CTIP1\|EVI9\|HBFQTL5\|ZNF856 | 53335 | 2p16.1 | 1 |
| EPOR | EPO-R | 2057 | 19p13.3-p13.2 | 1 |
| CD44 | CDW44\|CSPG8\|ECMR-III\|HCELL\|HUTCH-I\|IN\|LHR\|MC56\|MDU2\|MDU3\|MIC4\|Pgp1 | 960 | 11p13 | 1 |
| PAFAH1B2 | HEL-S-303 | 5049 | 11q23 | 1 |
| TCF12 | CRS3\|HEB\|HTF4\|HsT17266\|TCF-12\|bHLHb20 | 6938 | 15q21 | 1 |
| REL | C-Rel | 5966 | 2p13-p12 | 1 |
| EPC1 | Epl1 | 80314 | 10p11 | 1 |
| CNN3 | - | 1266 | 1p22-p21 | 1 |
| TAL2 | - | 6887 | 9q32 | 1 |
| PCSK7 | LPC\|PC7\|PC8\|SPC7 | 9159 | 11q23-q24 | 1 |
| MAST2 | MAST205\|MTSSK | 23139 | 1p34.1 | 1 |
| MUC1 | ADMCKD\|ADMCKD1\|CA 15-3\|CD227\|EMA\|H23AG\|KL-6\|MAM6\|MCD\|MCKD\|MCKD1\|MUC-1\|MUC-1/SEC\|MUC-1/X\|MUC1/ZD\|PEM\|PEMT\|PUM | 4582 | 1q21 | 1 |
| CEBPB | C/EBP-beta\|IL6DBP\|NF-IL6\|TCF5 | 1051 | 20q13.1 | 1 |
| CHST11 | C4ST\|C4ST-1\|C4ST1\|HSA269537 | 50515 | 12q | 1 |
| CEBPD | C/EBP-delta\|CELF\|CRP3\|NF-IL6-beta | 1052 | 8p11.2-p11.1 | 1 |
| WHSC1 | MMSET\|NSD2\|REIIBP\|TRX5\|WHS | 7468 | 4p16.3 | 1 |
| ZC3H12D | C6orf95\|MCPIP4\|TFL\|dJ281H8.1\|p34 | 340152 | 6q25.1 | 1 |
| FCGR2B | CD32\|CD32B\|FCG2\|FCGR2\|IGFR2 | 2213 | 1q23 | 1 |
| HOXA10 | HOX1\|HOX1.8\|HOX1H\|PL | 3206 | 7p15.2 | 1 |
| CEBPE | C/EBP-epsilon\|CRP1 | 1053 | 14q11.2 | 1 |
| ID4 | IDB4\|bHLHb27 | 3400 | 6p22.3 | 1 |
| SPIB | SPI-B | 6689 | 19q13.3-q13.4 | 1 |
| TENM2 | ODZ2\|TEN-M2\|TNM2\|ten-2 | 57451 | 5q34 | 1 |
| LHX4 | CPHD4 | 89884 | 1q25.2 | 1 |
| CCNE1 | CCNE\|pCCNE1 | 898 | 19q12 | 1 |
